# Review of Wearable Devices and Data Collection Considerations for Connected Health

**DOI:** 10.3390/s21165589

**Published:** 2021-08-19

**Authors:** Vini Vijayan, James P. Connolly, Joan Condell, Nigel McKelvey, Philip Gardiner

**Affiliations:** 1 Computing Department, Letterkenny Institute of Technology, F92 FC93 Letterkenny, Ireland; nigel.mckelvey@lyit.ie; 2 School of Computing, Engineering & Intelligent System, Ulster University Magee Campus, BT48 7JL Londonderry, Ireland; j.condell@ulster.ac.uk; 3 Rheumatology Department, Altnagelvin Hospital, Glenshane Road, BT47 6SB Londonderry, Ireland; pvgardiner@gmail.com

**Keywords:** wearable technology, digital healthcare, quantified self (QS), deep learning (DL), neural network (NN)

## Abstract

Wearable sensor technology has gradually extended its usability into a wide range of well-known applications. Wearable sensors can typically assess and quantify the wearer’s physiology and are commonly employed for human activity detection and quantified self-assessment. Wearable sensors are increasingly utilised to monitor patient health, rapidly assist with disease diagnosis, and help predict and often improve patient outcomes. Clinicians use various self-report questionnaires and well-known tests to report patient symptoms and assess their functional ability. These assessments are time consuming and costly and depend on subjective patient recall. Moreover, measurements may not accurately demonstrate the patient’s functional ability whilst at home. Wearable sensors can be used to detect and quantify specific movements in different applications. The volume of data collected by wearable sensors during long-term assessment of ambulatory movement can become immense in tuple size. This paper discusses current techniques used to track and record various human body movements, as well as techniques used to measure activity and sleep from long-term data collected by wearable technology devices.

## 1. Introduction

Wearable sensor technology has steadily grown in availability within a wide variety of well-established consumer and medical devices. Wearable devices can provide real-time feedback regarding a person’s health conditions; hence, they can provide an objective alternative to manage and monitor chronic disease progression, such as with the elderly, within rehabilitation, and for those with various disabilities [1]. Wearable sensors are widely used in healthcare, due to their hardware capacity, small footprint and lower cost compared to equivalent medical instruments capable of monitoring the same vital signs [2]. Furthermore, wearable technology decreases the cost of intensive treatment by allowing rehabilitation outside of the hospital in an ambulatory environment [3]. According to recent estimates, wearable technology will flourish over the next 25 years, resulting in a global cost savings of over $200 billion in the healthcare industry and a considerable reduction in clinician/patient interaction time [4]. Reports suggest that the number of wearable devices in use in 2020 was approximately 600 million, and current trends predict the number to increase to 928 million in 2021, and to reach 1100 million in 2022 [5].

Wearable technology offers many advantages within the healthcare environment but approved clinical devices have been slow to appear in healthcare settings. Data gloves and a ball with sensors have been used to track finger motions in stroke patients’ hand recovery therapy [6]. Wearable EMG sensors containing a sensor, electrodes, and Bluetooth Low Energy (BLE) communication technology can be used to assess nerve conduction, activation frequency, quantify and monitor electrical activity associated with muscle contractions and muscle response in injured tissue. Optical sensors that use a light-based technique to quantify the delicate magnetic fields produced by neurons firing in the brain may be used instead of MRI machines to create similar imaging, eliminating the expensive cooling or electromagnetic shielding required when patients require an MRI scan [4]. Additionally, the attachable skin format of these sensors improves the portability of the otherwise cumbersome devices, and can optimise bioelectrical signals obtained from users [7]. In patients with Chronic Obstructive Pulmonary Disease (COPD), self-management has been shown to increase the quality of life and reduce respiratory-related hospital admissions. Lightweight wireless pulse oximeters that detect oxygen levels in the blood by analysing real-time patient data are commonly used with COPD [8,9]. Apple has FDA approval for its ECG, as well as clearance in the European Economic Area, with more than 20 countries now able to make use of the health feature. Remote patient monitoring provided by wearable technology has also become an important tool throughout the Covid-19 pandemic. Wearables have been used in the UK to remotely monitor people with chronic disease or post-covid symptoms, and clinicians can remotely examine, regularly view vital sign data and provide adequate remote consultations [10].

Wearable devices are typically placed either directly on the wearer’s body, within clothing, or in semi-rigid structures, such as gloves, insoles, headwear and smartwatches. They can inter-communicate using the human body as a transmission channel [11] or through an appropriate transmission medium, such as BLE, Zigbee or Wi-Fi. Wearable devices capture, filter, and archive long-term physiological and activity data from the wearer. Due to their limited storage and computing capabilities, wearables may be unable to process data locally. As a result, they transfer captured data to a powerful remote computer or a cloud implementation, where the sensor information is deciphered, deconstructed, and results are meaningfully generated, interpreted and presented to the user [12]. Communication networks facilitate the intercommunication of sensors and control systems. Fifth generation (5G) communication technology improves broadband networks to support demanding requirements, such as always-available transmission services, low end-to-end latency, and a significant data rate boost over fourth generation (4G) communication technologies [13]. Digital healthcare uses this improved data transmission speed to enhance healthcare facilities, human health and ultimately population welfare.

**Figure 1:**
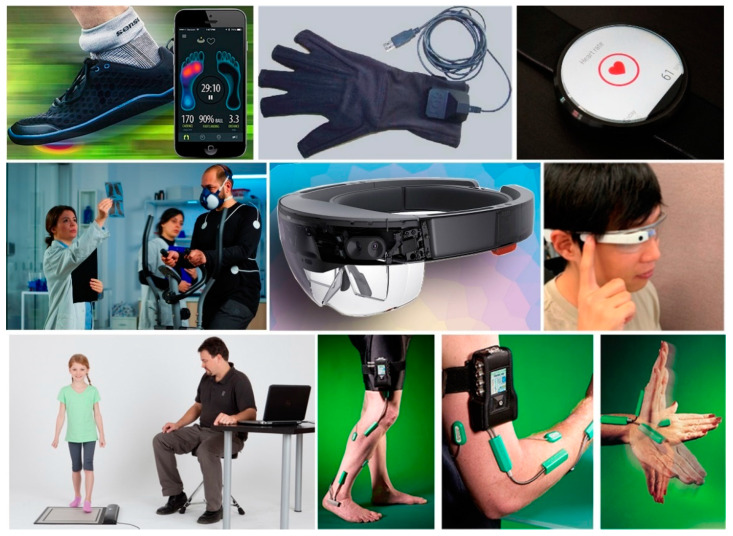
Various sensors can be attached to the body for motion capture, such as Smart Shoe, Pressure mat, sensors attached to leg, smartwatches, head sensors, data glove and Biometrics Goniometers and Torsiometers on toe and arm [15,16,17,18,19].

Automatic human activity recognition is an essential element in developing human-interaction applications in personal fitness and healthcare [14]. Fitness tracking measurements, such as step counts, distance covered, altitude climbed, rate of walking/running are all useful and are now well integrated into smartphones. Position sensing is also useful as the amount of sedentary time is a powerful predictor of health problems. Sleep sensing may also be done using movement sensors. Athletes have several specific sensor-based applications, and Inertial Measurement Unit (IMU) sensors are now widely deployed in professional sports [15]. The cost of these applications is also usually too high for regular use in the health service. Sensors typically used for detecting human activity are depicted in Figure 1. An accelerometer built into a smartwatch can track activity and sleep habits. Sensors positioned on the neck and lower back can collect data on the range of motion (ROM) of the head and upper body. Sensors may be attached to the leg postsurgical treatment to aid rehabilitation, and they are commonly used in athletics to track analytics, such as speed, velocity and long-term fitness progression. Headbands are used to collect data on head and neck motion. Data gloves are being tested at the research level for their effectiveness in capturing information on finger movements and tremors, as well as finger joint limitations. These gadgets are commercially available and are frequently utilised in fitness tracking and healthcare monitoring.

Wearable sensors are commonly used to assist in the transition of patient treatment from the clinic to the ambulatory environment. Their functionality can provide constant quantification of progress through treatment regimens by analysing patients’ prescribed physical activity routines for continuous improvement or decline [20]. Wearable devices have the advantage of providing objective data about movement and summarising this information in a way that clinicians can easily interpret. They can also be used in remote settings where access to health care is difficult (for example, on an oil rig) or when the patient is too ill or disabled to travel to the clinic/hospital. Furthermore, wearable data collected in an ambulatory environment is likely to provide a more accurate and representative measure of an individual’s physical status than a snapshot of data collected during a routine hospital appointment [21]. 

This review provides a detailed investigation of wearable devices and assessment techniques that are currently used in self-care and healthcare environments for monitoring device wearers, including those with musculoskeletal disorders. The paper begins with an overview of wearable technology and its applicability within a clinical environment. Typical healthcare sensor systems are investigated and discussed. Then self-care systems that are commonly used to quantify and monitor personal improvement are investigated. Measurement accuracy and other important wearable sensor discussions are then investigated, including device and data security. Finally, deep learning approaches and various algorithms for automated activity and sleep identification are discussed in detail. DL techniques may be able to detect and segment important objective data automatically within an ambulatory environment.

## 2. Wearable Technology in Clinical Trials

Research studies using IMU devices are becoming more common, but most are carried out in a controlled clinical environment under supervision. They focus on analysing multivariate sensor data to achieve unconstrained patient monitoring and extraction of patients’ physical and psychological conditions. Before a wearable device can be used for clinical or research purposes, it is important to understand the different types of data that may be required throughout the duration from a data collection perspective [22].

IMU sensors are most commonly used to measure and capture participant movement in a clinical environment. IMU’s are placed on specific locations of the patient’s body, such as the wrist, neck, back or waist [23], and the wearer’s motions are objectively observed and documented under the supervision of a physician or an experienced technical specialist. During a clinical assessment, patients may be assessed on specific functional tests that are relevant for the disease under scrutiny. Data collected from each sensor can then be used as additional information to further inform a clinician during a patient health status assessment of improvement or decline [24]. IMU sensors are widely used in motion tracking applications, such as altimeter sensors, gaming controllers, and Global Positioning System (GPS), and are used in a wide range of military and commercial use cases [25]. IMU movement data are usually stored in a controlling device, such as a microcontroller unit that controls data capture from the IMU sensor, can improve signal quality, and then implement a transmission protocol to forward sensor data to a connected third-party device, thus, permitting a posteriori data processing.

**Figure 2:**
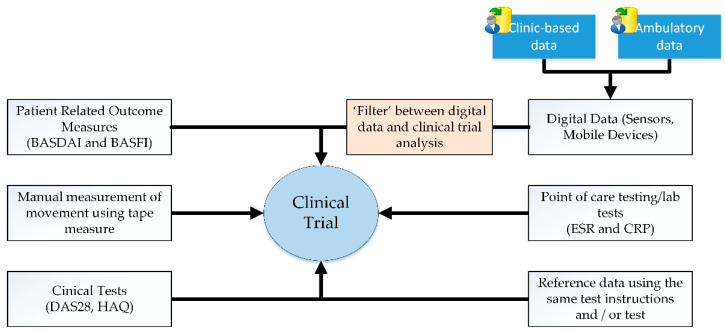
Various sources of data that may be used in a clinical trial.

In a clinical trial, participants wear sensors and perform various predefined movements with respect to the study protocol. To give an example, axial spondyloarthritis is a condition where changes in spinal mobility mark the progression of the disease—but up to now, clinicians have lacked an accurate way of monitoring this. Figure 2 shows the various types and sources of data that can be sourced and used within a clinical trial. Patient-related outcomes (PRO) refer to Health Assessment Questionnaires (HAQ) and Bath Ankylosing Spondylitis Functional Index (BASFI) scores and clinical tests, such as the Disease Activity Score (DAS28) and Bath Ankylosing Spondylitis Disease Activity Index (BASDAI) scores. These traditional clinical trial questionnaires and tests are used by physicians for musculoskeletal assessments, such as Rheumatoid Arthritis (RA) and Ankylosing Spondylitis (AS) [26]. Sensors or smartphone apps can capture digital data that corresponds to participant movements related to a clinical trial. The data may be clinical, or ambulatory based. A filter is typically used to minimise noise or unwanted data from sensor input signals. The inclusion of a reference movement or activity dataset can be used to process data obtained for a specific study or application and used for comparisons of representative movements, such as when using Artificial Intelligence (AI) techniques.

**Figure 3:**
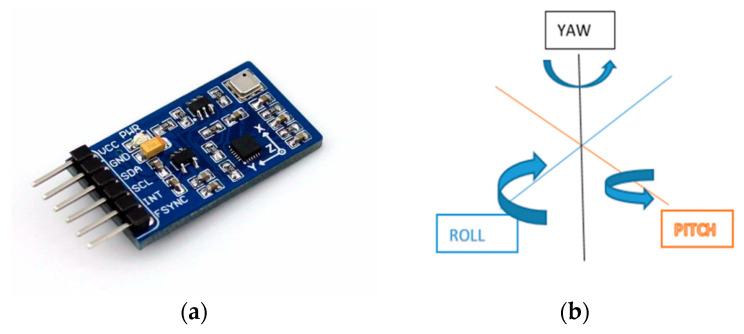
(**a**) IMU sensor chip and (**b**) DOF for IMU movement [31].

An IMU device contains an accelerometer, gyroscope and magnetometer that are combined to create independent measurements of the locations of an object to which it is attached [27]. Degrees of freedom (DOF) for an IMU sensor refers to the number of axes the sensor can measure to detect object orientation. Figure 3a depicts an IMU sensor and its controlling circuitry, and Figure 3b shows the 3 DOF measured by an IMU [28]. Data generated by an IMU may be incomplete, imprecise, or prone to error, due to (i) accelerometer data may be tainted by a gravitational force experienced by the IMU device [29], (ii) nearby magnetic disturbances may affect magnetometer measurements, and (iii) gyroscope data may drift due to unbounded time-varying factors [30].

The difference in IMU output values versus the expected output values is referred to as noise. The noise associated with IMU outputs owing to thermoelectrical reactions is known as random walk errors. Calibration errors are those that are caused by scale variables and orientations. The reaction of an accelerometer to rectification in the sensor is referred to as the vibration rectification error (VRE). It can cause a change in the offset value of the accelerometer reading [32]. When integrating sensor readings, the presence of these linear accelerations can cause orientation estimates to become corrupted during the sensor fusion process [33].

Using a complementary filter in conjunction with IMU devices can help to eliminate noise and reduce errors typically associated with IMU output data. Alternatively, a Kalman filter is frequently used with IMU sensors to enhance sensor output by eliminating various structural and ambient disturbances. To estimate the state of the IMU system, Kalman filters use mean square error minimisation, although they are unable to reduce vibration sounds caused by sensors. As a result, mechanical and certain software-based vibration filters have been designed to address this issue [34]. Complementary filters can be used with IMU sensors if there are two separate measurement sources for predicting the output variable and their values depend on the frequencies. Complementary filters typically include a high pass filter (HPF) and a low pass filter (LPF).

**Figure 4:**
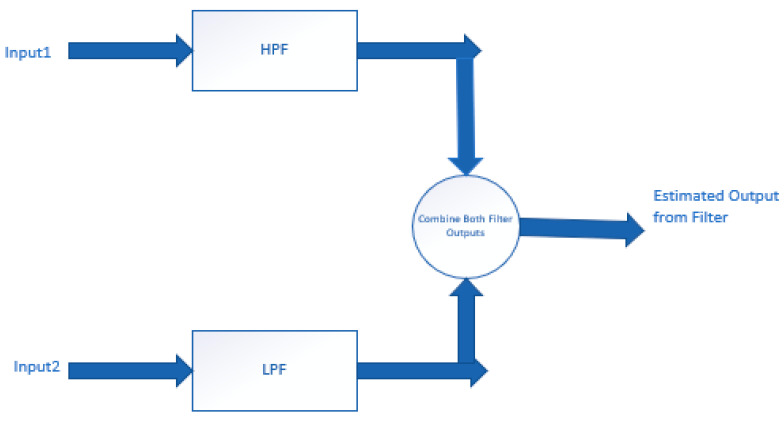
The architecture of a complementary filter.

The construction of a complementary filter is shown in Figure 4. In the diagram, input 1 and input 2 represent data from two different sources, such as three-axis readings from an IMU sensor, or alternatively, an accelerometer and gyroscope when sensor orientation is unimportant. This data contains inherent both low and high frequency noise. Input 1 contains data related to relatively low-frequency noise, whereas input 2 has data related to high-frequency noise. The LPF selects a higher cut-off frequency, and any data with frequencies less than that will be routed through that module. Similarly, an HPF configured with a lower cut-off value permits all data values above that. LPF and HPF cut-off settings are determined by the input data and application [6]. If given with correct cut-off frequencies, the predicted output of a complementary filter is practically noise-free and should enable quality data to flow through the filter [35]. IMUs are used in conjunction with complementary filters to produce high-quality input data for data processing when sufficient processing is not available to implement the computationally complex Kalman filter.

## 3. Wearable Devices in the Healthcare Environment

IMU sensors are commonly used for home monitoring of patient activity and recovery and are the typical sensor element used within a home monitoring system. A patient may be required to wear IMU sensors for longer than 24 h in any assessment session during an ambulatory monitoring scenario. During this period, the wearer is usually required to complete standardised functional assessments as part of their normal daily activities, whilst data are continually recorded from each wearable sensor. Therefore, rather than comprising only standardised functional test data, as would be the case in a clinical setting, the patient ambulatory movement dataset contains data corresponding to all patient movements whilst wearing each IMU sensor, including rest, casual walking, strenuous exercise and sleeping [36].

Clinical studies show that wearable devices are now being widely used to monitor activity at home to assess patients’ lifestyle concerns, obesity and disease symptoms, such as pulmonary conditions, diabetes, hypertension and cardiovascular disease. These are monitored by a suitably relevant wearable device which is commonly integrated with an IMU sensor and controlled with a smartphone application [37]. Wearable sensors are being used effectively to track musculoskeletal fitness. These sensors can accurately determine the angle of joint motion, neck movement, head rotation and flexion and extension movement [38]. Standardised functional tests are also effective ways of assessing musculoskeletal fitness by examining a person’s ability to perform tasks, such as bathing, dressing, feeding, grooming, mobility, stairs, toileting and transition without assistance. They are often used to validate human body functionality against standardised expected functionality values. Both assessment outcomes are used to estimate the patient’s degree of mobility. Devices capable of detailed functional assessment, such as the VICON and ViMove are efficient at collecting data related to standardised functional tests in a clinical setting or even in an ambulatory environment [39]. Table 1 provides a summary of various wearable technology devices are commonly used in a healthcare environment to quantify disease progression.

Traditional musculoskeletal disease detection methods depend on interpretation, questionnaires, and structured observations of patients [62]. Therefore, there exists the potential for a great deal of ambiguity during patient diagnosis. As an example, joint stiffness is a significant RA identifier and disease severity indicator that none of the traditional approaches can quantify. Wearable data gloves work on the principle of indirectly detecting the change in sensor angular rotation throughout a measurement session. A data glove equipped with IMU sensors can detect hand stability, and as a result, joint stiffness can be identified with reasonable precision. Machine Learning (ML) algorithms based on linear regression can also determine the disease seriousness of RA using a smartphone, reducing the need to visit a clinician. In a smartphone application, the device will train using the patients’ DAS28 and HAQ scores, as well as a self-assessed tender joint count (sTJC) and a self-assessed swollen joint count (sSJC). Patients’ trunk acceleration captured during walking can be measured with a smartphone application. This predictive model accounts for 67% of the DAS28 variance. It proposes that a smartphone program can accurately predict RA disease activity based on a non-invasive self-assessment of a combination of disabilities in everyday tasks, joint symptoms and walking ability [63].

**Figure 5:**
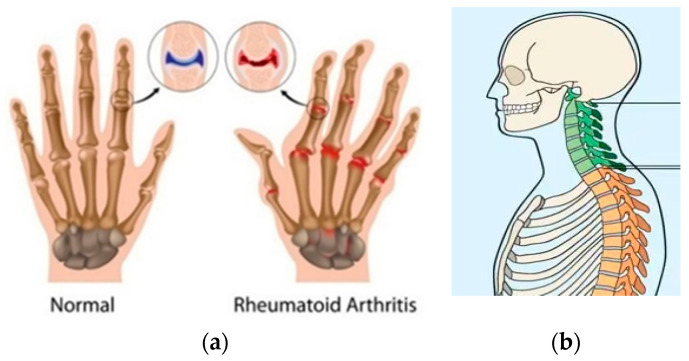
Musculoskeletal changes for (**a**) RA and (**b**) AS [64,65].

ASQOL is a metric that measures the quality of life in individuals with Ankylosing Spondylitis (AS) [64,65]. The musculoskeletal changes associated with RA and AS are shown in Figure 5. ASQOL is a measure of a person’s ability to fulfil the demands of daily life, while suffering from AS. This evaluation test requires answers about pain tolerance, sleep, relationships, independence and social life. These tests evaluate a patients’ peripheral and spinal joint pain, fatigue severity, morning stiffness, functional ability, axial position of the dorsal, lumbar, cervical spines, pelvic soft tissue, and hips, as well as localised tenderness. Patients, however, can encounter memory bias or incorrect self-judgments from outputs. Instrumented BASFI (iBASFI) adds a wearable accelerometer with activity measuring algorithms for evaluating performance-based measurements (PBM) [66]. It yields an index that describes clinically significant changes in different spinal movements in patients [67]. In patients with AS, IMUs are both reliable and valid in assessing spinal mobility. IMU sensors are attached to the collar, head, and lumbar spine to gather range of motion (ROM) data. The full ROM values calculated over a series of motions is used to calculate disease intensity. Thus, wearable sensors have been used to support clinicians during the diagnosis and rehabilitation of patients suffering from RA and AS.

The issue of using various health appraisal questionnaires is that (a) if an alternative is not available to patients, they can leave it null or N/A rather than entering details on what their opinion at that time (b) the pain scale cannot be used to accurately map pain magnitude and (c) all answers depend on a patient’s current physical and mental conditions which can be influenced by other factors, such as prescribed drugs or emotional state [68].

## 4. Wearable Devices for Quantified Self

The concept “quantified self” refers to the process of collecting personalised data about one’s own life and wellbeing using wearable devices and other advanced technology. Due to the prevalence of smartphones and sensor-rich wearable devices, data collection and analysis approaches are becoming more commonplace. Hence, the area of life logging and QS is rapidly expanding [69].

**Figure 6:**
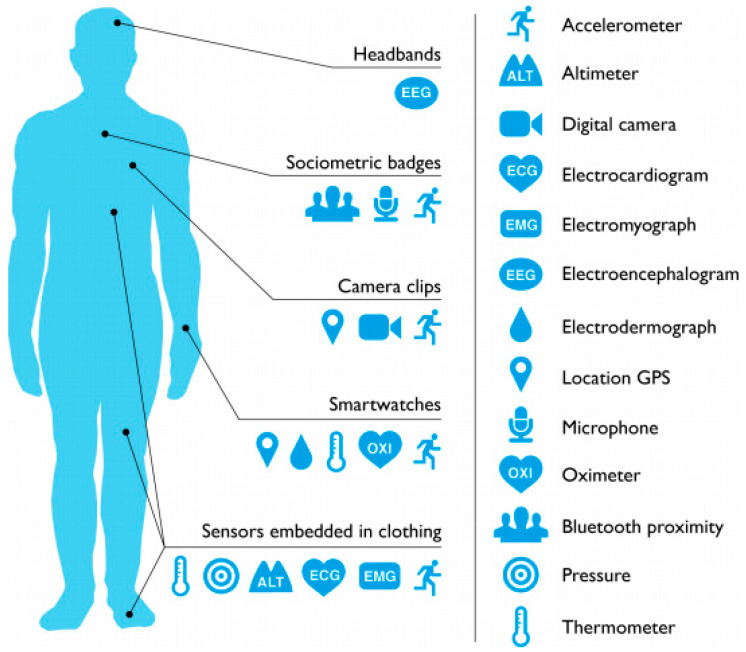
Wearable devices and their attached locations on the human body [71].

Wearable technology devices can assist the wearer in improving their sleep patterns, manage stress and increase productivity. If wearers are suffering from chronic disease conditions, these devices can provide sufficiently detailed data to monitor their disease progression [70]. Figure 6 shows a variety of wearable devices and their positioning on the human body. Headbands, sociometric badges, cameras, smartwatches and textile sensors are examples of commonly used wearable gadgets. These devices are integrated with various sensors that collect data from the human body.

Wearable devices, such as wristbands and associated mobile applications, can identify depression symptoms by monitoring vocal tones within phone conversations, measuring the duration of physical activity and sleep patterns. Basic fitness tracking functionalities in QS devices include step count, monitoring heart rate, counting calories burnt and gathering vital health data over time. Table 2 shows various smartwatches and activity trackers with their underlying technologies, common use and battery life. Such devices are typically used within the realm of QS.

The ability to accurately quantify movement dates back around 30 years with developing motion capture gait laboratories based upon techniques, such as optical photogrammetry in Vicon, magnetometry, such as Fastrack and force platform walkways, including GaitRite [86]. A variety of IMU sensor setups have been developed to study gait, but the optimal clinical analyses had not then been defined. Gait analysis has been quite widely investigated in patients with hip and knee osteoarthritis, but less so for RA and axSpA. The improvement in objective gait kinematics (such as asymmetry of gait) has become an important outcome measure that has led to improvements in patient care. Some clinicians advocate the use of standardised timed functional testing, such as the ‘five times ‘timed up-and-go test’ and the sit-to-stand’ test. These tests are regarded as a reliable measure of functional performance that capture transfer between legs, gait and turning movements. Formal assessments of walking ability are also important in a variety of chronic health conditions that affect gait. It is widely recognised that performance in these tests is strongly linked to functional ability and independence in the home [87].

Many tools support the assessment of various patient functional levels and ability and detect a person’s activity level throughout daily activities. Functional questionnaires and patient diaries are sometimes included in the analysis. They are subjective, require accurate patient recall, and usually cannot accurately reflect the functional ability of a patient at home. Specialist health practitioners can also carry out standardised functional tests with careful observation, but these measures are costly and time consuming, and the results are difficult to interpret by other members of a clinical team. Formal gait laboratory kinematic assessments are more accurate, but expensive, and availability is limited to a small number of specialist centres [88].

Wearable devices solve many of these limitations by using sensors for precise data acquisition, interpretation and prediction. The ePhysio, wearable system is designed to give remote monitoring and virtual coaching to patients who are completing rehabilitation tasks outside a clinical centre. It is equipped with IMUs and an Android program called Rehabilitation Hub that collects and processes data in real-time [89]. The RAPAEL Smart Glove19 by Neofect allows patients to rehabilitate their hands by wearing a glove and using associated technologies. This can be used to heal from injuries or to aid with complications that may occur as a result of neurological illnesses [90]. Wearable devices with cameras and motion sensors are used in a rehabilitation exercise evaluation for knee osteoarthritis, allowing the patient to self-manage rehabilitation progress. The accuracy for activity type categorisation was 97%, while the accuracy for exercise posture recognition was 88% [91]. Remote monitoring of workouts via sensors might allow experts to keep a close eye on a patient’s actions and development. The collection of data from everyday physical activities may also help clinicians to evaluate the progression of a condition more quickly and without the need for control sessions. Real-time monitoring of physical activities may also aid in delivering immediate feedback to the patient, while they do the activity, therefore boosting the quality of the rehabilitation. As a result, wearable sensors are widely being used in musculoskeletal healthcare assessments.

Data scientists can create new frameworks to assist with QS data processing, analysis and integration, as well as to take the lead in identifying open-access database tools and privacy requirements for how personal data are used. Next-generation QS implementations may provide methods for setting baselines and variability in objective measurements, making QS data relevant in behaviour improvement, using novel pattern recognition techniques and aggregating several self-tracking data streams from biosensors, wearable electronics, genomic data, cell phones and cloud-based services [92].

## 5. Measurement Accuracy

Accuracy refers to a sensor’s ability to produce a reading comparable to a “gold-standard” measuring device. The reproducibility of a calculation is referred to as its reliability. It denotes a sensor’s ability to detect and produce the same measurement multiple times, although the measurement may not be precise [93]. Wearable device validation entails demonstrating material validity, consistency, reliability and responsiveness to change. Technical requirements for wearable technology in healthcare applications include that they are fit-for-purpose for various use cases within the specific domain, can collect data in real-life environments, and are precise, consistent and tested in the healthcare community in which they would be utilised [94]. In addition, the type of data required for processing as part of healthcare applications or the applicability of sensors within a specific research study determines the relevant choice of suitable wearable sensors [95]. Table 3 compares the benefits of wearable technology applications to conventional healthcare monitoring systems. Wearable health monitoring devices outperform homecare and hospital environments in terms of scale, efficiency and ease of use.

Many wearable sensors, such as headbands and textile sensors, are effective in the short-term for activity tracking inside a controlled environment, but are inaccurate in the long-term [8]. Various wearable optical heart rate sensors have been shown in studies to be remarkably stable and accurate at rest and during sustained elevated heart rate. However, there are notable variances in how different devices respond to behavioural changes. Motion artefact is a suspected error with wrist-worn sensors, particularly during long-term heart rate monitoring [97]. It is commonly caused by sensor movement over the skin, temperature differences at the start and end of physical exercises, blood flow patterns, surface structure and thickness of the skin, and tissue density under the skin [98]. The accuracy of various wearable devices has been extensively discussed in the scholarly and scientific literature [99]. Table 4 shows various wearable sensors, their applications, technologies and the mean accuracy of each sensor type. Research indicates that wearable Inertial Motion Unit sensors ((IMUs) outperform other hardware sensors regarding the accuracy of measurements within a wide range of applications.

Wearable sensor noise is classified into two types: Motion-induced noise and sensor-intrinsic noise. Sensor implementations, such as those monitoring body movement and respiration rate, contain motion-induced noise. Sensor-intrinsic noise is typically caused by resistive sensor temperature noise and repetitive noise in capacitive sensors [108]. The sensor wearer may unintentionally cause errors throughout a lengthy initialisation or calibration procedure. Most wearable systems use wireless networking technologies to send captured data to a central processing system for analysis. The vulnerability of wireless communication channels can result in data loss during transmission, as well as errors in data analysis and prediction [109].

Almost all smartwatches and smartphones can now determine step count, with many systems reporting >95% accuracy. Various devices, such as MisFit Shine, Samsung Gear 1, Motorola Moto 360 and Apple Watch, are used to record step count and provide heart rate monitoring from the wearer. The accuracy and precision of wearable devices typically range from 92% to 99% [110]. Some technologies are more suited to indoor activities than others, due to restricted movements. Some devices can accurately detect step count, while others may be better for measuring heart rate. As a result, the accuracy of wearable devices varies depending on the device and application.

However, measurement accuracy in patients with low levels of activity is often lacking, and research indicates that when gait speed is slow and/or uneven, step count and activity determination are often unreliable. Research indicates a positive correlation between accuracy and the cost of wearable devices [111]. Table 5 demonstrates how the accuracy of step count and heart rate monitoring is affected by device cost. In this study, four participants completed each activity three times when wearing all smartwatches. Steps were manually calculated, and heart rate was measured with a pulse meter. After completing the activities, results from the smartwatches were compared to the pulse meter readings.

Smartwatches are movement sensitive. The tightness of the watch fit on the wearers arm is important for increased accuracy when measuring heart rate. Photoplethysmography (PPG)-based heart rate sensor outputs are influenced by varying degrees of tightness. Random user gestures will often confuse the predefined sensor calibration model, causing it to misread the user’s heart rate [113]. Micro Electromechanical System (MEMS) technology enables the design of compact, low power, low cost and high-performance wearable technology systems for a wide range of applications. The use of MEMS in fitness trackers attempts to strike a balance between energy consumption, performance, expense and size [114]. MEMS within Android devices attempts to reduce the data sampling rate to balance power consumption and efficiency. It is obvious that a high sampling rate contains more data than a low sampling rate, resulting in a hysteresis effect in the overall output [115]. The high sampling dataset includes more dynamic motions, which allows for a more effective filter design [116].

The wearable device’s main assessment problems are power consumption, connectivity capability, design constraints and security concerns. In today’s technology, the biggest obstacle in fabricating wearable and implantable devices is the powering process [117]. Since the communication area for wireless transmissions is normally restricted, the communication capability of wearable devices is reduced. Wearable interface security remains an unresolved problem. These devices jeopardise users’ privacy and security [118].

Despite their potential for use within clinical environments, wearable device accuracy and validity remain the most difficult challenges to overcome. Wearable applications must be easy to use and easily integrated into consumers’ daily lives if they are to be used for ongoing monitoring without causing disruption [119]. They should only require a minimal amount of charging in between measurement sessions. When wearable device computing and storage migrate to the cloud, health management solutions must be platform-independent, low-cost, universally available, and quickly deployable. Many off-the-shelf devices have immediate access to personalised monitoring software through cloud-based services [120].

## 6. Other Considerations for Wearable Technology 

The outcome of self-care or clinical trial evaluations is anticipated by quantifying data acquired by wearables. As a result, wearable device apps deal with a large amount of physiological data from the user. Therefore, the wearer and the captured sensor data from the sensor worn by the wearer are the two most important components of wearable device applications. Hence, the psychological elements of wearable devices, as well as data privacy and security, should be carefully considered. The psychological aspect of a wearable device includes the wearer’s comfort and mental wellbeing. Moreover, the data captured, stored and transmitted by a wearable device must be sufficiently private and secure, and must be stored and transferred with both traits in mind.

### 6.1. Psychological Aspects

Patients may be reluctant to use wearable technology devices that are uncomfortable to wear or that can be visually identifiable as wearable devices. Some users feel awkwardness and embarrassment when wearing an unusual looking sensor in public, such as when wearing a wristwatch alongside an activity tracker on the wrist or ankle. Epidermal biosensors, such as a graphene electronic tattoo (GET), are an emerging field in electronics that allows wearers to disguise sensor wearing from the public eye. This type of sensor resembles a tattoo, and is not distinguishable when worn, thus providing more confidence in using this type of wearable sensor to capture disease data [121]. Patients can experience trouble when charging wearable devices regularly, and this can cause emotional fatigue, due to the possibility that their wearable device could shut down. Important diagnostic data may be lost, due to no battery power. Larger batteries tend to reduce charging time and increase between-charging time, but they are uncomfortable to use, since they are larger in form size than smaller batteries and increase the overall dimensions of the wearable device [122].

Wearable sensors may also increase a wearer’s anxiety regarding their physical health. Elderly and physically unfit people who are keen to avail of detailed information regarding their general health conditions, such as heart rate, step speed and count, may attempt to complete physical exercises beyond their physical ability. It is quite common for wearers to follow fitness enhancement advice and recommendations from wearable devices without the guidance of clinical experts. Users may mistakenly correlate data from wearable devices with other diseases from which they are already suffering, giving them further emotional distress. Wearable devices inspire people to work out more, but they may leave them frustrated if they fail to meet the recommended activity level [123]. A retrospective analysis of data from wearable sensors used by chronic heart patients during self-care found that many participants wanted to learn more about their health and the possible connections between their daily activities and their disease status [124].

Wearers can use various mood tracking applications, such as MyTherapy, Breathe2Relax, MoodKit, MoodTracker or Daylio as part of QS [125]. Studies [126,127] show that those who use QS applications feel more in control over their mood, which helps them control their mood and show more confidence, a positive attitude and a better outlook towards their emotional wellbeing. Users can understand their emotional cycles by using mood-tracking applications. Then they can plan different strategies to improve happiness and manage stress. Changes in a person’s phone use pattern, regular everyday tasks and ordinary travel schedules where GPS data can help identify a change in a person’s mood. Mood-tracking apps play an important part in QS. A survey by the Institute of Health Matrix and Evaluation (IHME) estimated that more than one of every six individuals in European countries have a mental health problem [128]. Advancements in QS applications for mental wellbeing provide an important role for people who use them daily.

### 6.2. Data Privacy and Security

Data privacy and security must be considered within the context of wearable technology. Many wearable devices store data in local storage without encryption or data protection. As a result, there could be a high risk of losing confidential and personal health data. Wearable devices commonly connect to a smartphone using Bluetooth, NFC, or Wi-Fi. Unsecure wireless connectivity channels are insufficient to guard data against a brute-force attack [129]. Wearable sensors are always synchronised with smartphones for data transfer, and third-party apps installed on smartphones can increase the susceptibility to data hacking.

There are two types of data protection threats involved with wearable devices: Passive and active attacks. Passive attacks attempt to obtain the user’s password and personal information from the smart device. This technique does not damage or disrupt the target device. Active attacks, on the other hand, attempt to change or destroy the device. In a passive attack, a possible intruder can easily obtain data without the users’ knowledge, due to a lack of security on the wearable device communication pathway. Recent research indicated how a Fitbit device was subjected to data injection attacks, battery drain hacks and denial-of-service attacks [130]. Wearable devices are also exploited to obtain IMU data, including data from accelerometers, gyroscopes and magnetometers on fine-grained hand motions. This data can be used to execute Backward PIN-sequence inference algorithms to reproduce secret key entries to ATMs and electronic door locks [131].

Research demonstrates that most wearable device owners are concerned about the privacy of data stored within a wearable device [132]. Many fitness trackers save the user’s running, walking, or cycling routes by tracking GPS coordinates. This information could pose potential threats that could result in a breach of privacy. When switching GPS tracked locations between devices via Bluetooth or Wi-Fi, a privacy violation occurs [133]. Data protection threats can occur with integrated cameras and microphones in wearable devices. Microphones can trigger a privacy risk by capturing unauthorised audio or recording the voices of others without their consent. A wearable camera could also be hacked, exposing the user’s personal information and current surroundings. Many people can still use wearable systems to violate the privacy of others [118].

Since health information is sensitive or confidential in nature, data privacy and security are critical in healthcare applications that use wearable devices. To improve data protection and safety, users should be aware of the kind and volume of data captured by devices, as well as their potential significance. Other approaches to increase protection and privacy are to (i) use cryptographic mechanisms, such as a PIN to encrypt the device at all times, (ii) store data in cloud storage rather than local storage, which provides improved security and (iii) use secure network interfaces to migrate data from a wearable device to central storage [134]. It is impractical to use wearable devices with complete data protection. However, diligent use of these devices limits the likelihood of data loss.

## 7. Human Activity Detection Using Deep Learning Techniques

Human activity detection using deep learning (DL) techniques is a new field of innovation within wearable technology. Using DL techniques, a trained system thinks of the same intuition as a person does when identifying patterns for various activities. Pattern recognition and pattern matching techniques have been used to extract specific movement patterns from long-term datasets obtained by wearable sensor devices, especially those equipped with accelerometers and IMUs [135]. Pattern recognition techniques usually attempt to classify a dataset based on training and information extracted from previous patterns [136]. A pattern matching module checks for specific patterns amongst a large dataset, and results indicate whether searched patterns exist or not [137,138]. Pattern matching has also been used in a wide range of data science applications, such as Natural Language Processing, spam filtering, digital libraries and web search engines [139].

There are three types of ML algorithms: Supervised, semi-supervised and unsupervised. Supervised learning algorithms are trained by fully labelled datasets [140], and can then make predictions on new data [141]. Mapping inputs to outputs are predictive and can be evaluated through a well-defined system [142]. Supervised learning is mainly applicable for the classification of a discrete-class problem. The most important classifiers for supervised learning techniques are Decision trees, Bayesian Network (BN), Instance based learning (IBL) and Support Vector Machines (SVM) [143]. Offline Human Activity Recognition (HAR) systems produce results using supervised learning techniques. For example, supervised learning methods are used to train the machine in HAR applications that analyse eating patterns and exercise in patients suffering from diabetes, obesity and heart disease, as well as applications that measure the number of calories expended during an exercise regimen [144].

Unsupervised learning algorithms process unlabelled data and find patterns and relationships from them [145]. Data collected from mobile-based activity recognition applications are commonly defined as unsupervised. Smart environments integrated with a set of sensors generates heterogeneous data in terms of both semantics and format. Clustering is the primary strategy for creating a learning structure from unsupervised data collection. Data should be pre-processed for attribute extraction before implementing the clustering algorithm [146]. System training for semi-supervised learning system combines small quantities of a fully labelled dataset with large quantities of unlabelled data [147]. Semi-supervised learning techniques are used to train unlabelled data to a recognition model for applications, such as feature extraction, pattern recognition and speech recognition. Semi-supervised learning techniques are implemented using multigraphs, which propagate labels through a graph containing both labelled and unlabelled data [148]. DL is a subset of ML which can learn from unsupervised data and provides a system to learn from those datasets [149]. Continuous human movement detection and analysis is a tough problem in ML, since it is difficult to define the characteristics and duration of various movement patterns. Human motion characteristics vary from person to person. Each person’s motions of body components, such as the arm, leg and head for regular activities, such as walking, jogging and eating, are unique. The time required to do each of these activities also varies between individuals. As a result, generating patterns for each activity to train the ML system is a major undertaking. With the advancement of DL technology, manual feature extraction is no longer necessary, and performance improvement in complicated human activity identification is possible [150].

An Artificial Neural Network (ANN) based classifier performs well in real-time gesture identification using IMU data inputs [151]. To extract movement data from a sensor-enabled smartphone, pattern recognition algorithms are used [152]. Human motion detection and categorisation from IMU sensors have been used to aid in sporting activities. They are also implemented utilising pattern recognition and an ANN [153]. Pattern recognition algorithms are also employed in face recognition systems, where patterns for diverse facial configurations are retrieved and examined for pattern matching in order to perform the facial recognition process [154]. An ANN system trained on clinical datasets for pattern identification and matching is capable of automatically distinguishing standardised functional data patterns from datasets containing long-term movement patterns [155]. If an ANN system can automatically detect standardised functional tests of human movement from long-term data, then the sensors could provide a long-term measurement of patient activity and recovery at home with minimal intervention [156].

HAR based on data outputs from wearable sensors is a new research area within the field of Body Area Networks (BAN) and ubiquitous computing. Applications of online HAR systems are highly applicable within healthcare environments. For example, monitoring patients with mental or physical pathologies are essential for their protection, safety and recovery. eWatch is the most significant online HAR technology. An eWatch usually includes four sensors: A light sensor, an accelerometer, a microphone and a thermometer. They demonstrated accuracy ranging from 70% to 90% for six different ambulatory tasks [157]. Vigilante is a HAR-capable Android application. This program is linked to a chest sensor, which collects patient physiological data. For three ambulatory behaviour identification applications, they achieved an average accuracy of 92.6% [158]. Tapia et al. is another HAR system that generates 80.6% accuracy for 30 activities, including lifting weights, doing push-ups and rowing [159]. Finally, Kao is a HAR device that incorporates a triaxial accelerometer. It assessed seven activities: Driving, biking, punching, brushing teeth, knocking, swinging and using a computer. It has a total accuracy of 94.71% [160]. OpenHealth is an open-source platform for health monitoring. This includes a wearable device, standard software interfaces and reference implementations of human activity and gesture recognition applications. Experiment results demonstrate that the system achieves greater than 90% accuracy for all actions, such as stand, jump, walk and sit. When the user performs a gesture, the OpenHealth wearable device is mounted on the user’s wrist and records accelerometer data. The OpenHealth software program identifies movements, such as up, down, left and right by using a NN classifier. Trials with seven users show that the wearable device can identify these movements with an accuracy of 98.6% [161]. WaistonBelt X is a belt-style wearable device with sensing and intervention capabilities. This approach attempts to assist health behaviour change to prevent health issues caused by poor lifestyle behaviours, such as inactivity and poor body posture. Seven activities were trailed with 17 participants: Lying down, sitting, standing, walking, walking down and upstairs, and running. WaistonBelt X received an F1 score of 0.82, with some misclassifications occurring between sitting and standing, walking and walking up and down stairs [162].

HAR research often uses various statistical ML tools and techniques to manually construct new features and extract existing ones from various movement patterns. Statistical learning methods have been widely used to analyse and find solutions for different activity identification problems [163]. The nature of the sensors used for activity recognition (either external or wearable) makes the criterion to classify HAR systems. Hybrid approaches are an efficient class of systems that intend to exploit both techniques [164].

Naive Bayes (NB) and K-Nearest Neighbour (KNN) classifiers are often used to recognise seven human body motions, including jumping, walking and running [165]. HAR can extract traits that can accurately discriminate between different activities. Transform coding, and symbolic representation of raw data are two more popular feature extraction approaches in human activity identification research; however, they employ an average of previously extracted features and need expert knowledge to build new ones. In recent years, the recognition and popularity of DL approaches have impacted their incorporation into HAR applications [147].

Various NN architectures are better suitable for movement categorisation than others. A study that collects data for movement data classifications using a Nexus One Android smartphone integrated with accelerometer sensor devices. The data were fed into two DL algorithms: Convolutional Neural Network (CNN) and Long Short-Term Memory-Recurrent Neural Network (LSTM-RNN). The findings of CNN and LSTM-RNN are compared to those of classic classifiers, such as k-NN and Feed Forward Neural Network. The accuracy of a TensorFlow-based Convolution Neural Network (CNN) in classifying movements, such as walking patterns, limps and foot placement, was 84% [166]. The CNN exceeded the LSTM in terms of developing an ideal Deep Q Network (DQN) for pattern recognition of human arm movement and gesture recognition using DL methods and wearable-sensor technologies [167]. Non-parametric discovery of human movement patterns for walking, sedentary activities, running and jumping from accelerometer data using a Hierarchical Dirichlet process (HDP) model implemented using Support Vector Machine (SVM) exhibited precision of 0.81 and recall of 0.77 for sliding window time durations. However, studies have shown that an HDP model cannot estimate activity levels automatically over lengthy periods of time [135]. Apriori and Pattern Recognition (PR) algorithms were used to represent tracking and forecasting patterns of a moving item in a wireless sensor network. The model’s accuracy was determined by comparing actual graph data to predicted graph data [168], and results demonstrated the PR algorithm indicated better prediction than the Apriori algorithm. The PR-Algorithm and Apriori Algorithm Standard Deviations were around 2.6 and 3.32, respectively. CNN were used to build HAR-based motion detection utilising U-Net. U-Net is a data segmentation architecture based on a CNN system that improves the sliding window approach in data segmentation. Research [169] employed four distinct datasets in their research, including WISDM, UCI HAPT, UCI OPPORTUNITY and the self-collected “Sanitation” dataset. Simple accuracy and F-scores were used to calculate prediction accuracy. The U-Net technique outperformed SVM, Decision Tree, CNN, LSTM and CovLSTM (architecture combines two convolutional layers and two LSTM layers with 32 hidden units) in terms of accuracy and F-Score. The accuracy of the U-Net approach is up to 94.7% in a dataset with a significant number of short-term actions. The F-Score for the Sanitation dataset was 0.998 for Run and 0.905 for a walk. CNN obtained an F-score of 0.78 for walking.

InnoHAR, a deep neural network model for complex human activity identification, was created in 2019 by combining Recurrent Neural Network (RNN) with inception neural network architectures constructed using Keras. On a sliding window dataset, InnoHAR demonstrated significantly higher performance and strong generalisation performance than state-of-the-art and baselines. This model has been generalised and validated using three datasets: Opportunity, PAMAP2, and Smartphone, with F-scores of 0.946, 0.935 and 0.945, respectively [170]. DL models based on a mixture of recurrent and CNN neural networks were developed for automatically recognising athletic tasks using wearable sensor data. Deep neural networks trained on optical and IMU data obtained nearly identical F1 scores between 0.8 and 0.9. According to this study, integrating more body parts increased the classification accuracy of their NN system [171].

Using integrating inertial sensor data and visuals, a Deep NN implemented by a CNN was investigated for recognising multimodal human motions. Their study looked at the outcomes of three distinct types of input data: IMU only, the camera only and a combination model that accepted IMU and camera inputs, as well as the classification network for matching inputs. The cross-validated accuracy for action classification showed that the combination setting outperformed camera-only and IMU-only systems. According to the findings, a NN system based on IMU sensor data can help physicians in situations when action recognition approaches based on cameras fail to reliably forecast the precise human position [172]. Combining Simple Recurrent Units (SRUs) with Gated Recurrent Units (GRUs) of a NN resulted in developing a Hybrid DL model for human activity identification utilising multimodal body sensing data. The F1-score estimated for deep SRUs-GRUs models is greater than the F1-score generated for current models. This increase in efficiency is due to the system’s capacity to process and retain patterns that recognise human activities from multimodal body sensing data. This shows that pattern recognition algorithms may be used to identify human activities [173]. Human action recognition utilising wearable sensors and a neural network based on the Akamatsu Transform employing CNN were also developed effectively and efficiently. This approach recognises human behaviours based on data acquired from wearable sensors and learnt using a suitable NN. The Akamatsu transform is a method for extracting characteristics from sensor data. It reached an accuracy of more than 85% [174]. Table 6 shows numerous techniques employed within wearable technology studies for autonomously monitoring human activity, including the technologies employed, the number of participants, and the model accuracy.

Other ML approaches, such as SVM, Hidden Markov Model (HMM), and linear regression, have also been used to detect HAR from wearable sensor data. For monitoring the balancing abilities of patients or the elderly with impaired balance, an ANN system based on single inertial sensor data were created. During walking, this system calculated Inclination Angle (IA) and Center Of Mass–Center Of Pressure (COM-COP) characteristics for various participants. The devised method assessed individuals with Scoliosis, Cerebral Palsy and Parkinson’s Disease’s capacity to balance. Feed-forward ANN and Long-Short-Term Memory (LSTM) network models were created independently, and the root-mean-square error (rRMSE) for each model was determined separately. The Feed-forward ANN had a 15% error rate, whereas the LSTM exhibited a 9% improvement in accuracy [175].

**Table 6:** Comparison of various methods to automatically detect human activity monitoring using wearable sensor technology.

Ref	ML Model/NN Type	Details	Epochs	No. of Participants	Test for Analysis	Results
[166]	(CNN) and (LSTM-RNN)	TensorFlow is used to implement the NN.	40	22	Accuracy (84%)	CNNs may perform better than LSTM-RNN for real-time datasets.
[167]	CNN with the Deep Q Neural Network (DQN) model compared with LSTM models and DQN	CCR, EER, AUC, MAP and the CMC.		50	Classification accuracy (98.33%)	CNN model performing better than the LSTM model.
[176]	1-D Convolutional neural network (1-D CNN)—a RNN model with LSTM	3+3 C-RNN designed for data processing.	1000	80	Accuracy (90.29%)	Model works well for lower sampling rates. However, for large data set accuracy is getting lower.
[135]	Hierarchical Dirichlet process (HDP) model to detect human activity levels	SVM		27	Precision of 0.81 and recall of 0.77.	(HDP) model that can infer the number of levels automatically from a sliding window time duration.
[168]	Apriori Algorithm and Pattern Recognition (PR) Algorithm	New algorithm for PR is designed and implemented in MATLAB.		9	Standard deviation of Predicted v/s Actual Graph (Standard Deviations were around 2.6 for PR-Algorithm and 3.32 for Apriori algorithm).	PR algorithm indicated better prediction than the Apriori algorithm.
[177]	Hierarchical Dirichlet Process Model (HDPM)	Feed forward neural network.	50	201	Simple accuracy (sitting—78.60%, standing—9.45%, walking—26.87%)	The physical activity levels are automatically learned from the input data using the HDPM.
[169]	HAR method based on U-Net	CNN	100	266,555 samples and 5026 windows	Accuracy and Fw-score (Max. Accuracy of 96.4% and Fw-Score of 0.965).	U-Net method overcomes the multiclass window problem inherent in the sliding window method and realises the prediction of each sampling point’s label in time series data.
[170]	InnoHAR—DL model	Combination of inception neural network and RNN structure built with Keras.		9	Opportunity, PAMAP2, and Smartphone datasets with F-scores of 0.946, 0.935 and 0.945, respectively.	Consistent superior performance and has good generalisation performance.
[171]	Deep Neural Network	Combination of convolutional and recurrent NN.		417	F1-Score in between 0.8–0.9 for different activities.	Simulated sensor data demonstrates the feasibility of classifying athletic tasks using wearable sensors.
[172]	Deep Neural Network	Fully connected CNN.	50	5 (20 actions per person)	cross validated accuracy for action classification. (Camera only—85.3% IMU only 67.1%, Combined—86.9%).	Action recognition algorithm utilising both images and inertial sensor data that can efficiently extract feature vectors using a CNN and performs the classification using an RNN.
[173]	Hybrid DL model	Combines the simple recurrent units (SRUs) with the gated recurrent units (GRUs) of neural networks.	50	1007	Accuracy (99.8%)	Deep SRUs-GRUs networks to process the sequences of multisensors input data by using the capability of their internal memory states and exploit their speed advantage.
[174]	CNN	Akamatsu Transform		120	Accuracy (85%)	Proposed a human action recognition method using data acquired from wearable sensors and learned using a Neural Network.
[178]	SVM, ANN and HMM, and one compressed sensing algorithm, SRC-RP	DL using MATLAB.		4 people with 5 different tests	Recognition accuracy for different datasets (Debora—93.4%, Katia—99.6%, Wallace—95.6%).	Three different ML algorithms, such as SVM, HMM and ANN, and one compressed sensing-based algorithm, SRC-RP are implemented to recognise human body activities.
[179]	ML	Ensemble Empirical Mode Decomposition (EEMD), Sparse Multinomial Logistic Regression algorithm with Bayesian regularisation (SBMLR) and the Fuzzy Least Squares Support Vector Machine (FLS-SVM).		23	Classification accuracy (93.43%).	A novel approach based on the EEMD and FLS-SVM techniques is presented to recognise human activities. Demonstrated that the EEMD features can make significant contributions in improving classification accuracy.
[180]	ML	WEKA		30	Accuracy (98.5333%)	Sensors on a smartphone, including an accelerometer and a gyroscope were used to gather and log the wearable sensing data for human activities.
[151]	Real-time Gesture Pattern Classification	Neural network-based classifier model.		1040	Accuracy (77%)	Human hand gesture recognition using manually collected data and processed by LSTM layer structure. Accuracy is denoted using unity visualisation.
[181]	Pattern Recognition Methods for Head Gesture-Based Interface of a Virtual Reality Helmet (VRH) Equipped with a Single IMU Sensor	Classifier uses a two-stage PCA-based method, a feedforward artificial neural network, and random forest.		975 gestures from 12 patients	Classification rate (0.975)	VRH with sensors are used to collect data. Dynamic Time Warping (DTW) algorithm used for pattern recognition.
[182]	Hand Gesture Recognition (HGR) System.	Restricted Coulomb Energy (RCE) neural networks distance measurement scheme of DTW.		252	Accuracy (98.6%)	Hand Gesture Recognition (HAR) system for Human-Computer Interaction (HCI) based on time-dependent data from IMU sensors.
[183]	Motion capturing gloves are designed using 3D sensory data	Classification model with ANN.		6700	Accuracy (98%)	Data gloves with IMU sensors are used to capture finger and palm movements.
[184]	Quaternion-Based Gesture Recognition Using Wireless Wearable Motion Capture Sensors	SVM and ANN		11	Accuracy (90%)	Multisensor motion capturing system that is capable of identifying six hand and upper body movements.

To recognise people’s emotional states from entire body motion patterns, a feedforward deep convolution neural network model was constructed [185]. Based on data from a single wearable IMU, the ML system was designed to identify age-related and surface changes in walking. DL with LSTM was used to complete system training, prediction and implementation. Four models were trained using all sensor data from an IMU vs. accelerometer, gyroscope and magnetometer data only. The results revealed that completely trained models for surface and age with complete IMU data had good precision (96.4, 95.2%), recall (96.3, 94.7%), f1-score (96.3, 94.6%) and accuracy (96.3, 94.6%). (96.3, 94.7%) [186]. 

ML algorithms have already been used to identify movement patterns. The researchers used a VRH and incorporated IMU sensors to assess head gesture position. Data from 975 head movement patterns from 12 people were used as input data for feedforward ANN, a two-stage PCA-based approach and a random forest. The system, which comprised of VRH and integrated IMUs, achieved 91% accuracy [181].

Using Restricted Coulomb Energy (RCE) neural networks, a real-time Hand Gesture Recognition (HGR) system for Human-Computer Interaction (HCI) based on time-dependent input from IMU sensors was developed. Every neuron in RCE NN has a central point and a radius. The system attempted to determine the central point and radius of each neuron throughout the training phase. The mechanism determined the distance factor between various neurons throughout the pattern recognition process. This data were coupled with input feature values to decide which neuron would be activated next. This sort of technology had a 98.6% accuracy rate [182]. To record finger joint flexibility and hand movements, data gloves with integrated IMU sensors were employed. Data gloves collect information and transmit it wirelessly and in real-time to a compatible receiving device. With 0.1 s of predicting, the system reached a speed of 8.94 milliseconds per frame and an accuracy of 98% [183]. Using this data glove system, a multi-sensor motion capture system capable of identifying six motions was constructed. Participants’ hand and upper body movements were used to collect data. The motion identification module was created using SVM and ANN. Gestures as a jab, upper cut, throw, raise, block and sway were detected with greater than 85% accuracy [184].

Automatic pattern recognition for various activities and sleep states accurately is a milestone in wearable device research. It has many applications in healthcare, sports and personal care. Many studies use data from wrist-worn accelerometers determine the activity level and sleep level of individuals [187]. Polysomnographic (PSG) pattern recognition for automated classification of sleep-waking stages in neonates is implanted using EEG, electrooculogram (EOG) and EMG. The system records sleep data from EEG, ocular movements from EOG and muscular tone from EMG. The automated system’s performance was tested indirectly by analysing different sleep phases in newborns with 70% accuracy [188]. PSG data are used to create a pattern recognition algorithm for multiclass sleep stage analysis. ANN with sigmoidal and softmax transfer functions is used to classify different phases of sleep. The Bayesian network evaluates the probability of several classes. The system’s mean classification accuracy was 88.7% [189]. Linear regression is efficient and applicable for finding cause and effect relationships between input and output variables. There are different libraries and algorithms available to implement pattern recognition techniques using the linear regression concept [190], and it is commonly used to correlate personal health factors with sleep quality by analysing data collected from wearable sensors. Multivariate analyses show that poor sleep scores cause more medical expenses and depressive symptoms [191].

## 8. Algorithms for Activity and Sleep Recognition

**Figure 7:**
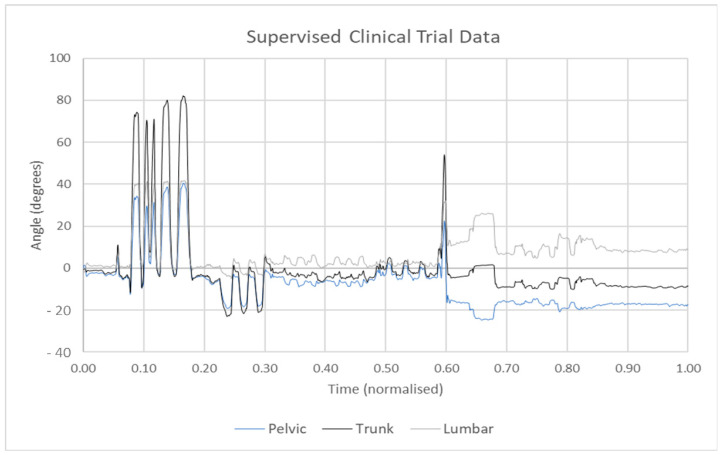
Sensor data were collected for several ranges of movement tests. The data contains random noise and movement for several tests, which require segmentation and extraction before analysis can occur.

Data collected during an occasional clinical assessment represents a very limited snapshot of a patient’s physiological condition. Decisions about the patient’s health condition, status of disease and recommended types of treatments and medical procedures are concluded by comparing data recorded during a clinical assessment to general population averages. These decisions may not always be relevant and tailored to the individual. The extrapolation of such a snapshot should be extended to a longer period for generating valid inferences about a patient’s health. Automatically extracting activity and sleep patterns from long-term datasets can aid to increase diagnostic accuracy [24]. Sensor data can, therefore, be used to extrapolate activity and sleep measurement using relevant software techniques and libraries. Figure 7 shows data from a supervised clinical trial for the identification of the range of movement tests. The X-axis represents normalised time, whereas the Y-axis represents angular values of movement in degrees. The motions in the red box represent the real movements, whereas the data in the black box are noises. The algorithm for automatically detecting activities should be fine-tuned to distinguish patterns of expected activities from alternative activities or rest periods that are considered noise. The time required to accomplish each activity varies by gender, age and health condition. As a result, developing an efficient automated activity detection system is a difficult task.

GGIR is a widely used R package used to process and extract physical activity from multiday raw accelerometer data, and for sleep research. Regardless of brand-specific accelerometer units, data are entered into GGIR in m/s^2^ or gravitational acceleration. GGIR estimates the degree of physical activity, idleness and sleep based on accelerometer data. It comes with lots of built-in functions and arguments for analysing data from wearable sensors. In terms of input data, it may provide output summaries for several levels of activity, such as personal, daily and night. Unfortunately, only a small body of evidence exists to indicate that raw accelerometer data are an accurate representation of body acceleration and most scientific evidence indicates that the validity of accelerometer data are dependent mostly on epoch averages [192]. In 52 children, the GGIR algorithm classifies seven distinct types of activities with 91% accuracy for hip-worn accelerometers and 88% accuracy for wrist-worn accelerometers [193].

Cole–Kripke is an algorithm used to devise a sleep score. This algorithm was initially tested for adult populations ranging from 35 to 65 years of age [194]. The algorithm calculates a Sleep Index (SI) value with respect to levels of physical activity. If the SI is less than 1, the sleep state is deemed awake (W), otherwise, it is regarded as sleeping (S). The Cole–Kripke algorithm is sensitive because genuine sleep state is typically predicted as asleep (s); however, research suggests that [195] Cole–Kripke sleep misclassification was high in research involving children as compared to adults. Previous research indicates that this algorithm undervalued sleep and exaggerated wakefulness in children and vice versa in adults. Both GGIR and Cole–Kripke algorithms do not provide facilities to segment data based on specific movement patterns.

An in-home sleep study of 40 healthy people measuring sleep characteristics using Motionlogger^®^ Micro Watch Actigraphy (MMWA) using Cole–Kripke algorithm, revealed 94% to 98% sensitivity and 42% to 54% specificity in identifying Sleep epochs [196]. Another research study for in-home polysomnography (PSG) using the Cole–Kripke algorithm on a sample of 35 healthy volunteers found that it was more sensitive to identify sleep (88–96%), but less specific to detect awake time (35–64%) [195]. ActiGraph Link’s sleep-monitoring feature was tested on 49 adults. Both accelerometers’ epochs were categorised as sleep or awake, and the data were transformed to total sleeping duration, wake after sleep beginning and sleep efficiency. Cole–Kripke’s algorithm’s sleep/wake state was determined to be 96.13% effective [197]. Studies suggest that the Cole–Kripke algorithm is effective in extracting sleeping patterns in adults.

If all human activities can be extracted automatically from ambulatory gestures, patient monitoring effort can be considerably reduced. This could prove to be a significant leap in wearable technology applications, and ultimately, in the medical area.

## 9. Conclusions

Human movement analysis is an interesting area of research and is growing in popularity, due to the increase in the implementation of wearable technology within ambulatory and home care environments. Wearable devices can enable users to assess their own health. Analysing and identifying specific types of activity within sensor data can further enhance the assessment of the patient’s quality of daily life. This can be achieved by analysing a wide variety of sensor variables using various techniques. Wearable sensors can passively collect information about a patient’s daily activities, and hence. These data are rich in objective detail when compared to clinical assessment data. The increased availability of sensor data allows clinicians to gain closer insights into a patient’s health than is possible using traditional subjective methods. This review examined various wearable devices used for QS, clinical assessments and automated monitoring of activity and sleep patterns. Current studies employing DL approaches to automatically detect certain activity or sleeping patterns from data received from wearable technologies are also examined.

One of the main purposes of wearable devices is to create and develop precise, accurate and unambiguous results. Research in this area requires completing explicitly quantifiable work packages, including hardware validation assessment, development and testing of a clinical protocol, and implementation and evaluation of a feasibility study. Future advancements in this area will provide clinicians with more detailed information on various movements contained in data recorded at home. This data will provide users with a better awareness of their health and clinicians with a more granular assessment of the patient’s fitness, as well as specific insights into physical mobility.

## Figures and Tables

**Table 1:** Wearable devices that are used in the healthcare environment to quantify disease progression.

Wearable Device	Body Location	Typical Use Case/Disease Condition	Captured Movement
Wearable Cutaneous Haptic Interface (WCHI) [40]	Finger	Parkinson’s Disease	Three degrees of freedom to measure disease conditions, such as tremor and bradykinesia.
Smart Electro-Clothing Systems (SeCSs) [41]	Heart	Health Monitoring	Surface electromyography (sEMG); HR, heart rate variability.
Xsens DOT [42]	All over the body	Healthcare, sports	Osteoarthritis
5DT data glove [43]	Fingers and wrist	Robust Hand Motion Tracking	Fibre optic sensors measure flexion and extension of the Interphalangeal (IP), metacarpophalangeal (MCP) joints of the fingers and thumb, abduction and adduction, and the orientation (pitch and roll) of the user’s hand.
Neofect Raphael data glove [44]	Fingers, wrist and forearm	Poststroke patients	Accelerometer and bending sensors measuring flexion and extension of finger and thumb.
Stretchsense data glove [45]	Hand motion capture	Gaming, augmented and virtual reality domains, robotics and the biomedical industries.	Flexion, extension of fingers and thumb.
Flex Sensor (Data glove) [46]	Finger	Rheumatoid Arthritis (RA), Parkinson’s disease and other neurological conditions/rehabilitative requirements.	Flexion and extension of the (IP), (MCP) joints of the fingers and thumb and the abduction and adduction movements.
X-IST Data Glove [47]	Hand and fingers	Poststroke patients	Five bend sensors and five pressure sensors measure MCP, PIP finger and thumb movement.
MoCap Pro (Smart Glove) [48]	Hand and fingers	Stroke	Capture bend of each MCP and proximal interphalangeal (PIP) joint.
Textile-Based Wearable Gesture Sensing Device [49]	Elbow and knee	Musculoskeletal disorders	Flexion angle of elbow and knee movements
VICON system [50]	Shoulder and elbow	Musculoskeletal disorders	Humerothoracic, scapulothoracic joint angles and elbow kinematics.
Goniometer-Pro [51]	Knee	Stroke	Passive flexion of knee.
Smart Garment Sensor System [52]	Leg	Strain sensor	Lower limb joint position analysis.
Fineck [53,54]	Neck	Monitor neck movements and respiratory frequency.	Flexion-extension and axial rotation repetitions, and respiratory frequency.
SMART DX [55]	All over the body	Gait clinical assessment and multifactorial movement analysis.	Dynamic analysis of muscle activity, postural analysis, motor rehabilitation.
ViMove [56]	Neck, lower back and knee	Movement and Activity Recognition in sports and clinical monitoring.	Flexion-extension and axial rotation.
Dubbed Halo [57]	Wrist	Voice monitoring application called ‘Tone’.	Detect the “positivity” and “energy” from the human voice.
Polysomnography sensors [58]	Chest, hand, leg and head	Identify sleep apnoea	Breathing volume and heart rate.
Pulse oximetry [59]	Finger	Pulmonary disease	Monitor oxygen saturation, respiratory rate, breathing pattern and air quality.
TZOA [60]	Textile	Respiratory disease	Measure air quality and humidity.
Eversense Glucose Monitoring, Guardian Connect System and Dexcom CGM [61]	Hand	Diabetes	Glucose level monitoring.

**Table 2:** Various wearable devices are commonly used for QS. Battery life indicates the number of days available to capture data before recharging the onboard battery.

Wearable Device for QS	Type	Technology Used	Well Known Applications	Battery Life
Apple Watch [72]	Smartwatch	IMU, Blood oxygen sensor, electrical heart sensor, optical sensors.	Basic fitness tracking, Blood Oxygen Level, ECG, step count, sleep patterns.	1 day
Fitbit Sense [73]	Smartwatch	IMU, blood oxygen sensor, electrical heart sensor, optical sensors, temperature sensor, electrodermal sensor.	Basic fitness tracking, stress management, SpO2, skin temperature, sleep and FDA-cleared ECG, tracking electrodermal activity.	6 days
Samsung Gear2 [74]	Smartwatch	IMU, electrical heart sensor.	Basic fitness tracking.	<1 day
Samsung GearS [75]	Smartwatch	IMU, electrical heart sensor.	Basic fitness tracking.	<1 day
iHealth Tracker (AM3) [76]	Fitness Tracker	IMU	Steps taken, calories burned, distance travelled, sleep hours and sleep efficiency.	5–7 days
Pebble Watch [77]	Smartwatch	IMU, ambient light sensor.	Cycling app to measure speed, distance and pace through GPS.	3–6 days
Mi Band 6 [78]	Fitness Tracker	IMU, PPG heart rate sensor, capacitive proximity sensor.	Heartrate measurements, sleep tracking, sport tracking.	14 days
MisFit Shine [79]	Fitness Tracker	IMU, capacitive touch sensor.	Tracks steps, calories, distance, automatically tracks light and deep sleep, activity tagging feature for any sports.	4–6 months
Sony Smartwatch 4 (SWR10) [80]	Smartwatch	GPS, IMU, optical heart rate sensor and altimeter.	Distance and duration of workout, heart rate monitoring, steps count	2–4 days
Fitbit Flex [81]	Fitness Tracker	IMU, heart monitor, altimeter.	Track steps, sleep and calories.	Up to 5 days
Decathlon ONCoach 100 [82]	Activity Tracker	GPS, IMU, altimeter	Step count, track light and deep sleep, record the start and the end of a sport session, average speed and distance and calories consumed.	6 months
Actigraphy [83]	Activity recognition/Sleep pattern recognition	IMU	Inclination, gait analysis, fall detection, sleep quality analysis.	14 days
Garmin VivoSmart HR+ [84]	Activity recognition/Sleep analysis	IMU, heartrate monitor altimeter, GPS	Steps, distance, calories, floors climbed, activity intensity and heart rate.	8 h
MotionNode Bus [85]	Motion tracking	miniature IMU	Motion tracking using IMU data.	7 h

**Table 3:** Requirements of wearable health monitoring devices within various healthcare services [96].

Medical Service	Place of Care	Required Sensor Performance and Accuracy		Requirements
		Healthcare Use	Self-Monitoring	
**Domiciliary care**	Patient’s home	High	Medium	Portable, robust, ease of use
**Hospital care**	Hospital environment	High	Medium	Portable within a hospital setting, high accuracy
**Wearable health monitoring**	Anywhere, Any time	Medium	Medium	Small and light, highly portable and unobtrusive

**Table 4:** Measurement accuracy and use cases for various wearable systems.

Wearable Sensor	Usage	Sensor Technology	Reported Accuracy
Grid-eye [100]	Human tracking or detection	Temperature sensing using Infrared radiation	80%
Wearable Biochemical Sensors [101]	Detect biomarkers in biological fluids	Physicochemical transducer	95%
Wearable Biophysical Sensors [102]	Detect biophysical parameters, such as heartrate, temperature and blood pressure	Sensor electrodes	94%
Adhesive patch-type wearable sensor [103]	Monitoring of sweat electrolytes	Radio-frequency identification (RFID)	96%
Tattoo-Based Wearable Electrochemical Devices [104]	Monitor fluoride and pH levels of saliva	Body-compliant wearable electrochemical devices on temporary tattoos	85%
RFID Tag Antenna [105]	Tracking of patients in a healthcare environment	RFID	99%
Pedar system [106,107]	Human gait analysis	Pressure sensors capture insole-based foot pressure data	88%
Wearable IMU Sensor [21,22,23]	Motion tracking, activity tracking, gait altitude, fall detection	Accelerometer, Gyroscope, Magnetometer	99%

**Table 5:** Accuracy of step count and heart rate monitoring for smartwatches. Information adapted from [112].

Metric	Smartwatch	Accuracy (Steps)		Typical Cost (May 2021)
		200 Steps	1000 Steps	
Step count	Apple Watch	99.1%	99.5%	€480
	MisFit Shine	98.3%	99.7%	€185
	Samsung Gear 1	97%	94%	€150
Heart rate measurement	Apple Watch	99%	99.9%	€480
	Motorola Moto 360	89.5%	92.8%	€110
	Samsung Gear Fit	93%	97.4%,	€150
	Samsung Gear 2	92.3%	97.7%	€130
	Samsung Gear S	91.4%	89.4%	€110
	Apple iPhone 6 (with cardio application	99%	99.2%	€180

## Data Availability

Not applicable.

## References

[1] Pantelopoulos A., Bourbakis N.G. (2009). A Survey on Wearable Sensor-Based Systems for Health Monitoring and Prognosis. IEEE Trans. Syst. Man. Cybern. Part C Appl. Rev..

[2] Liu S. (2019). Wearable Technology–Statistics & Facts Statista. www.statista.com/topics/1556/wearable-technology/.

[3] Frost B. (2014). Market Research Report. Strategy R.

[4] Pando A. (2019). Wearable Health Technologies and Their Impact on the Health Industry. Forbes. www.forbes.com/sites/forbestechcouncil/2019/05/02/wearable-health-technologies-and-their-impact-on-the-health-industry/#4eac185f3af5.

[5] Tankovska H. (2020). Global Connected Wearable Devices 2016–2022 Statista. Statista. www.statista.com/statistics/487291/global-connected-wearable-devices/.

[6] Connolly J. Wearable Rehabilitative Technology for the Movement Measurement of Patients with Arthritis. Ulster University, February 2015. https://ethos.bl.uk/OrderDetails.do?did=1&uin=uk.bl.ethos.675471.

[7] Song M.-S., Kang S.-G., Lee K.-T., Kim J. (2019). Wireless, Skin-Mountable EMG Sensor for Human–Machine Interface Application. Micromachines.

[8] Massaroni C., Saccomandi P., Schena E. (2015). Medical Smart Textiles Based on Fiber Optic Technology: An Overview. J. Funct. Biomater..

[9] Jouffroy R., Jost D., Prunet B. (2020). Prehospital pulse oximetry: A red flag for early detection of silent hypoxemia in COVID-19 patients. Crit. Care.

[10] Best J. (2021). Wearable technology: Covid-19 and the rise of remote clinical monitoring. BMJ.

[11] Vijayan V., McKelvey N., Condell J., Gardiner P., Connolly J. Implementing Pattern Recognition and Matching techniques to automatically detect standardized functional tests from wearable technology. Proceedings of the 2020 31st Irish Signals and Systems Conference (ISSC).

[12] Majumder S., Mondal T., Deen M.J. (2017). Wearable Sensors for Remote Health Monitoring. Sensors.

[13] Sun H., Zhang Z., Hu R.Q., Qian Y. (2018). Wearable Communications in 5G: Challenges and Enabling Technologies. IEEE Veh. Technol. Mag..

[14] Schrader L., Toro A.V., Konietzny S., Rüping S., Schäpers B., Steinböck M., Krewer C., Müller F., Güttler J., Bock T. (2020). Advanced Sensing and Human Activity Recognition in Early Intervention and Rehabilitation of Elderly People. J. Popul. Ageing.

[15] Cha J., Kim J., Kim S. (2019). Hands-free user interface for AR/VR devices exploiting wearer’s facial gestures using unsupervised deep learning. Sensors.

[16] (2021). Sensoria Fitness: Motion and Activity Tracking Smart Clothing for Sports and Fitness. https://store.sensoriafitness.com/.

[17] TEKSCAN Gait Mat|HR Mat|Tekscan. Photo Courtesy of Tekscan™, Inc. www.tekscan.com/products-solutions/systems/hr-mat.

[18] Image Courtesy 5DT.com; DT Technologies Home—5DT. https://5dt.com/.

[19] NEXGEN NexGen Ergonomics–Products–Biometrics–Goniometers and Torsiometers. www.nexgenergo.com/ergonomics/biosensors.html.

[20] Bell (2019). J. Wearable Health Monitoring Systems.

[21] Lee J., Kim D., Ryoo H.-Y., Shin B.-S. (2016). Sustainable Wearables: Wearable Technology for Enhancing the Quality of Human Life. Sustainability.

[22] Hurdles C. (2017). Applied Clinical Trials- Your Peer-Reviewed Guide to Gobal Clinical Trials Management. https://cdn.sanity.io/files/0vv8moc6/act/346a82766960a17da2099b5f1268d0efba485d2a.pdf.

[23] Grimm B., Bolink S. (2016). Evaluating physical function and activity in the elderly patient using wearable motion sensors. EFORT Open Rev..

[24] Dhairya K. (2018). Introduction to Data Preprocessing in Machine Learning. https://towardsdatascience.com/introduction-to-data-preprocessing-in-machine-learning-a9fa83a5dc9.

[25] Mavor M.P., Ross G.B., Clouthier A.L., Karakolis T., Graham R.B. (2020). Validation of an IMU Suit for Military-Based Tasks. Sensors.

[26] Balsa A., Carmona L., González-Alvaro I., Belmonte M.A., Tena X., Sanmartí R. (2004). Value of Disease Activity Score 28 (DAS28) and DAS28-3 Compared to American College of Rheumatology-Defined Remission in Rheumatoid Arthritis. J. Rheumatol..

[27] Callmer J. (2016). Autonomous Localization in Unknown Environments. Master’s Thesis.

[28] Estevez P., Bank J., Porta M., Wei J., Sarro P., Tichem M., Staufer U. (2012). 6 DOF force and torque sensor for micro-manipulation applications. Sens. Actuators A Phys..

[29] Pandey A., Mazumdar C., Ranganathan R., Tripathi S., Pandey D., Dattagupta S. (2008). Transverse vibrations driven negative thermal expansion in a metallic compound GdPd3B0.25C0.75. Appl. Phys. Lett..

[30] Sparkfun (2016). Accelerometer, Gyro and IMU Buying Guide–SparkFun Electronics. www.sparkfun.com/pages/accel_gyro_guide.

[31] On Board Mpu9255 10 Axial Inertial Navigation Module10 Dof Imu Sensor(b)gyroscope Acceleration Sensor–Buy Mpu9255 10 Axial Inertial Navigation Module, 10 Dof Imu Sensor, Gyroscope Acceleration Sen. www.alibaba.com/product-detail/on-board-MPU9255-10-axial-inertial_60838848689.html.

[32] CANAL GEOMATICS IMU Accuracy Error Definitions Canal Geomatics. http://www.canalgeomatics.com/.

[33] Ahmed H., Tahir M. (2017). Improving the Accuracy of Human Body Orientation Estimation with Wearable IMU Sensors. IEEE Trans. Instrum. Meas..

[34] Guner U., Canbolat H., Unluturk A. Design and implementation of adaptive vibration filter for MEMS based low cost IMU. Proceedings of the 2015 9th International Conference on Electrical and Electronics Engineering (ELECO).

[35] Paina G.P., Gaydou D., Redolfi J., Paz C., Canali L. (2011). Experimental comparison of kalman and complementary filter for attitude estimation. Proc. AST.

[36] De Arriba-Pérez F., Caeiro-Rodríguez M., Santos-Gago J.M. (2016). Collection and Processing of Data from Wrist Wearable Devices in Heterogeneous and Multiple-User Scenarios. Sensors.

[37] Iman K., Al-Azwani H. (2016). Integration of Wearable Technologies into Patient’s Electronic Medical Records. Qual. Prim. Care.

[38] Lawton E.B. (1969). ADL and IADL treatment; Assessment of older people: Self-maintaining and instrumental activities of daily living. Gerontologist.

[39] Camp N., Lewis M., Hunter K., Johnston J., Zecca M., Di Nuovo A., Magistro D. (2020). Technology Used to Recognize Activities of Daily Living in Community-Dwelling Older Adults. Int. J. Environ. Res. Public Health.

[40] Lee Y., Kim M., Lee Y., Kwon J., Park Y.-L., Lee D. (2018). Wearable Finger Tracking and Cutaneous Haptic Interface with Soft Sensors for Multi-Fingered Virtual Manipulation. IEEE/ASME Trans. Mechatron..

[41] Sayem A.S.M., Teay S.H., Shahariar H., Fink P.L., Albarbar A. (2020). Review on Smart Electro-Clothing Systems (SeCSs). Sensors.

[42] Xsens (2015). Home Xsens 3D Motion Tracking. www.xsens.com/.

[43] Caeiro-Rodríguez M., Otero-González I., Mikic-Fonte F.A., Llamas-Nistal M. (2021). A Systematic Review of Commercial Smart Gloves: Current Status and Applications. Sensors.

[44] Smart Glove Neofect. www.neofect.com/us/smart-glove.

[45] Shen Z., Yi J., Li X., Lo M.H.P., Chen M.Z.Q., Hu Y., Wang Z. (2016). A soft stretchable bending sensor and data glove applications. Robot. Biomimetics.

[46] Henderson J., Condell J., Connolly J., Kelly D., Curran K. (2021). Review of Wearable Sensor-Based Health Monitoring Glove Devices for Rheumatoid Arthritis. Sensors.

[47] De Pasquale G. (2018). Glove-based systems for medical applications: Review of recent advancements. J. Text. Eng. Fash. Technol..

[48] Djurić-Jovičić M., Jovičić N.S., Roby-Brami A., Popović M.B., Kostić V.S., Djordjević A.R. (2017). Quantification of Finger-Tapping Angle Based on Wearable Sensors. Sensors.

[49] Shyr T.-W., Shie J.-W., Jiang C.-H., Li J.-J. (2014). A Textile-Based Wearable Sensing Device Designed for Monitoring the Flexion Angle of Elbow and Knee Movements. Sensors.

[50] Mjøsund H.L., Boyle E., Kjaer P., Mieritz R.M., Skallgård T., Kent P. (2017). Clinically acceptable agreement between the ViMove wireless motion sensor system and the Vicon motion capture system when measuring lumbar region inclination motion in the sagittal and coronal planes. BMC Musculoskelet. Disord..

[51] Ortiz M., Juan R., Val S.L. (2017). Reliability and Concurrent Validity of the Goniometer-Pro App vs. a Universal Goniometer in determining Passive Flexion of Knee. Int. J. Comput. Appl..

[52] Totaro M., Poliero T., Mondini A., Lucarotti C., Cairoli G., Ortiz J., Beccai L. (2017). Soft Smart Garments for Lower Limb Joint Position Analysis. Sensors.

[53] Veari Presents Fineck Smart Wearable Device for Neck Health. www.designboom.com/technology/veari-fineck-smart-wearable-device-neck-health-11-25-2014/.

[54] Lo Presti D., Carnevale A., D’Abbraccio J., Massari L., Massaroni C., Sabbadini R., Zaltieri M., Bravi M., Sterzi S., Schena E. (2020). A Multi-Parametric Wearable System to Monitor Neck Computer Workers. Sensors.

[55] BTS Products Applications_BTS Bioengineering. https://www.btsbioengineering.com/applications/.

[56] ViMove2 Analyse Patient Movement & Muscle Activity–DorsaVi EU. www.dorsavi.com/uk/en/vimove/.

[57] Amazon’s New Fitness Tracker Halo Will Monitor Your Tone of Voice—Quartz. https://qz.com/1897411/amazons-new-fitness-tracker-halo-will-monitor-your-tone-of-voice/.

[58] Schätz M., Procházka A., Kuchyňka J., Vyšata O. (2020). Sleep Apnea Detection with Polysomnography and Depth Sensors. Sensors.

[59] Liebling S., Langhan M. (2018). Pulse Oximetry. Nurs. Times.

[60] Aliverti A. (2017). Wearable technology: Role in respiratory health and disease. Breathe.

[61] Kumar H.S. (2019). Wearable Technology in Combination with Diabetes. Int. J. Res. Eng. Sci. Manag..

[62] Baig M.M. (2017). Early Detection and Self-Management of Long-Term Conditions Using Wearable Technologies. Ph.D. Thesis.

[63] Nishiguchi S., Ito H., Yamada M., Yoshitomi H., Furu M., Ito T., Shinohara A., Ura T., Okamoto K., Aoyama T. (2014). Self-Assessment Tool of Disease Activity of Rheumatoid Arthritis by Using a Smartphone Application. Telemed. e-Health.

[64] Managing Rheumatoid Arthritis–NPS MedicineWise. www.nps.org.au/consumers/managing-rheumatoid-arthritis.

[65] How Is a Person Affected by Ankylosing Spondylitis (AS)_ _ SPONDYLITIS. https://spondylitis.org/about-spondylitis/possible-complications/.

[66] Swinnen T.W., Milosevic M., Van Huffel S., Dankaerts W., Westhovens R., De Vlam K. (2016). Instrumented BASFI (iBASFI) Shows Promising Reliability and Validity in the Assessment of Activity Limitations in Axial Spondyloarthritis. J. Rheumatol..

[67] Irons K., Harrison H., Thomas A., Martindale J. (2016). Ankylosing Spondylitis (Axial Spondyloarthritis). The Bath Indices. www.nass.co.uk.

[68] Annoni F. (2000). The health assessment questionnaire. J. Petrol..

[69] Rawassizadeh R., Momeni E., Dobbins C., Mirza-Babaei P., Rahnamoun R. (2015). Lesson Learned from Collecting Quantified Self Information via Mobile and Wearable Devices. J. Sens. Actuator Netw..

[70] Çiçek M. (2015). Wearable Technologies and Its Future Applications. Int. J. Electr. Electron. Data Commun..

[71] Piwek L., Ellis D., Andrews S., Joinson A. (2016). The Rise of Consumer Health Wearables: Promises and Barriers. PLoS Med..

[72] Whitney L. (2020). 21 Tips Every Apple Watch Owner Should Know PCMag. PCMag. www.pcmag.com/how-to/20-tips-every-apple-watch-owner-should-know.

[73] Fitbit Sense In-Depth Review_All the Data Without the Clarity_DC Rainmaker. www.dcrainmaker.com/2020/09/fitbit-sense-in-depth-review-all-the-data-without-the-clarity.html.

[74] Stein S. Samsung Gear 2 Review_A Smartwatch that Tries to Be Everything–CNET. www.cnet.com/reviews/samsung-gear-2-review/.

[75] https://www.google.com/search?client=firefox-b-d&q=smartdevice-samsung-gear-s-um+.

[76] Activity W., Tracker S., Guide Q.S. Wireless Activity and Sleep Tracker. https://uk.pcmag.com/migrated-99802-smartwatches/122576/21-tips-every-apple-watch-owner-should-know.

[77] PEBBLE-WATCH BLUETOOTH Watch User Manual Pebble Technology. https://fccid.io/RGQ-PEBBLE-WATCH/User-Manual/user-manual-1868584.

[78] Xiaomi Mi Band 6 User Manual Download (English Language). www.smartwatchspecifications.com/xiaomi-mi-band-6-user-manual/.

[79] Mannion P. Teardown: Misfit Shine 2 and the Art of Power Management. EDN. https://www.edn.com/teardown-misfit-shine-2-and-the-art-of-power-management/.

[80] Apps and Fitness–Sony Smartwatch 3 Review TechRadar. www.techradar.com/reviews/sony-smartwatch-3/4.

[81] Bennett B. (2016). Fitbit Flex Review_A Most Versatile, Feature-Packed Tracker–CNET. www.cnet.com/reviews/fitbit-flex-review/.

[82] ONcoach 100. https://support.decathlon.co.uk/oncoach-100.

[83] ActiGraph Link. https://actigraphcorp.com/actigraph-link/.

[84] Garmin VivoSmart HR+. https://www.expansys.jp/garmin-vivosmart-hr-regular-size-black-taiwan-spec-291780/.

[85] MotionNode Bus Wearable Sensor Network. www.motionnode.com/bus.html.

[86] Wilson S., Laing R.M. Wearable Technology: Present and Future. Proceedings of the 91st World Conference.

[87] Bohannon R.W., Bubela D.J., Magasi S.R., Wang Y.-C., Gershon R.C. (2010). Sit-to-stand test: Performance and determinants across the age-span. Isokinet. Exerc. Sci..

[88] Maenner M.J., Smith L.E., Hong J., Makuch R., Greenberg J.S., Mailick M.R. (2013). Evaluation of an activities of daily living scale for adolescents and adults with developmental disabilities. Disabil. Heal. J..

[89] Vallati C., Virdis A., Gesi M., Carbonaro N., Tognetti A. (2018). ePhysio: A Wearables-Enabled Platform for the Remote Management of Musculoskeletal Diseases. Sensors.

[90] Rodgers M.M., Alon G., Pai V.M., Conroy R.S. (2019). Wearable technologies for active living and rehabilitation: Current research challenges and future opportunities. J. Rehabil. Assist. Technol. Eng..

[91] Chen K.-H., Chen P.-C., Liu K.-C., Chan C.-T. (2015). Wearable Sensor-Based Rehabilitation Exercise Assessment for Knee Osteoarthritis. Sensors.

[92] Swan M. (2013). The Quantified Self: Fundamental Disruption in Big Data Science and Biological Discovery. Big Data.

[93] Hessing T. Measurement Systems Analysis (MSA) Six Sigma Study Guide. https://sixsigmastudyguide.com/measurement-systems-analysis/.

[94] Tim D. (2018). Getting the Most out of Wearable Technology in Clinical Research. J. Clin. Stud..

[95] Patringenaru I. (2015). Temporary Tattoo Offers Needle-Free Way to Monitor Glucose Levels. http://ucsdnews.ucsd.edu/pressrelease/temporary_tattoo_offers_needle_free_way_to_monitor_glucose_levels.

[96] Yamada I., Lopez G. Wearable sensing systems for healthcare monitoring. Proceedings of the 2012 Symposium on VLSI Technology (VLSIT).

[97] Zhang Y., Song S., Vullings R., Biswas D., Simões-Capela N., Van Helleputte N., Van Hoof C., Groenendaal W. (2019). Motion Artifact Reduction for Wrist-Worn Photoplethysmograph Sensors Based on Different Wavelengths. Sensors.

[98] Bent B., Goldstein B.A., Kibbe W.A., Dunn J.P. (2020). Investigating sources of inaccuracy in wearable optical heart rate sensors. NPJ Digit. Med..

[99] Piccinini F., Martinelli G., Carbonaro A. (2020). Accuracy of Mobile Applications versus Wearable Devices in Long-Term Step Measurements. Sensors.

[100] Size S., For S., Detection I. (2016). Grid-Eye State of the Art Thermal Imaging Solution. https://eu.industrial.panasonic.com/sites/default/pidseu/files/whitepaper_grid-eye.pdf.

[101] Schrangl P., Reiterer F., Heinemann L., Freckmann G., Del Re L. (2018). Limits to the Evaluation of the Accuracy of Continuous Glucose Monitoring Systems by Clinical Trials. Biosensors.

[102] Stanley J.A., Johnsen S.B., Apfeld J. (2020). The SensorOverlord predicts the accuracy of measurements with ratiometric biosensors. Sci. Rep..

[103] Rose D.P., Ratterman M.E., Griffin D.K., Hou L., Kelley-Loughnane N., Naik R.R., Hagen J.A., Papautsky I., Heikenfeld J.C. (2014). Adhesive RFID Sensor Patch for Monitoring of Sweat Electrolytes. IEEE Trans. Biomed. Eng..

[104] Bandodkar A.J., Jia W., Wang J. (2015). Tattoo-Based Wearable Electrochemical Devices: A Review. Electroanalysis.

[105] How Accurate Can RFID Tracking Be RFID Journal. www.rfidjournal.com/question/how-accurate-can-rfid-tracking-be.

[106] De Castro M.P., Meucci M., Soares D., Fonseca P., Borgonovo-Santos M., Sousa F., Machado L., Vilas-Boas J.P. (2014). Accuracy and Repeatability of the Gait Analysis by the WalkinSense System. BioMed Res. Int..

[107] Weizman Y., Tan A.M., Fuss F.K. (2018). Accuracy of Centre of Pressure Gait Measurements from Two Pressure-Sensitive Insoles. MDPI Proc..

[108] Mohd-Yasin F., Nagel D.J., Korman E.C. (2009). Noise in MEMS. Meas. Sci. Technol..

[109] Yu Y., Han F., Bao Y., Ou J. (2015). A Study on Data Loss Compensation of WiFi-Based Wireless Sensor Networks for Structural Health Monitoring. IEEE Sensors J..

[110] ElAmrawy F., Nounou M.I. (2015). Are Currently Available Wearable Devices for Activity Tracking and Heart Rate Monitoring Accurate, Precise, and Medically Beneficial?. Health Inform. Res..

[111] Pardamean B., Soeparno H., Mahesworo B., Budiarto A., Baurley J. (2019). Comparing the Accuracy of Multiple Commercial Wearable Devices: A Method. Procedia Comput. Sci..

[112] Mardonova M., Choi Y. (2018). Review of Wearable Device Technology and Its Applications to the Mining Industry. Energies.

[113] Ra H.-K., Ahn J., Yoon H.J., Yoon D., Son S.H., Ko J. I am a “Smart” watch, Smart Enough to Know the Accuracy of My Own Heart Rate Sensor. Proceedings of the 18th International Workshop on Mobile Computing Systems and Applications.

[114] Ciuti G., Ricotti L., Menciassi A., Dario P. (2015). MEMS Sensor Technologies for Human Centred Applications in Healthcare, Physical Activities, Safety and Environmental Sensing: A Review on Research Activities in Italy. Sensors.

[115] Bieber G., Haescher M., Vahl M. Sensor requirements for activity recognition on smart watches. Proceedings of the 6th International Conference on PErvasive Technologies Related to Assistive Environments.

[116] Khoshnoud F., De Silva C.W. (2012). Recent advances in MEMS sensor technology-mechanical applications. IEEE Instrum. Meas. Mag..

[117] Ghomian T., Mehraeen S. (2019). Survey of energy scavenging for wearable and implantable devices. Energy.

[118] Ching K.W., Singh M.M. (2016). Wearable Technology Devices Security and Privacy Vulnerability Analysis. Int. J. Netw. Secur. Appl..

[119] Byrom B., Watson C., Doll H., Coons S.J., Eremenco S., Ballinger R., Mc Carthy M., Crescioni M., O’Donohoe P., Howry C. (2018). Selection of and Evidentiary Considerations for Wearable Devices and Their Measurements for Use in Regulatory Decision Making: Recommendations from the ePRO Consortium. Value Health.

[120] Patel S., Park H., Bonato P., Chan L., Rodgers M. (2012). A review of wearable sensors and systems with application in rehabilitation. J. Neuroeng. Rehabil..

[121] Ameri S.K., Hongwoo J., Jang H., Tao L., Wang Y., Wang L., Schnyer D.M., Akinwande D., Lu N. (2017). Graphene Electronic Tattoo Sensors. ACS Nano.

[122] Chandel V., Sinharay A., Ahmed N., Ghose A. Exploiting IMU Sensors for IOT Enabled Health Monitoring. Proceedings of the First Workshop on IoT-Enabled Healthcare and Wellness Technologies and Systems.

[123] Healthcare-in-Europe Smart Watches and Fitness Trackers Useful but May Increase Anxiety. https://healthcare-in-europe.com/en/news/smart-watches-fitness-trackers-useful-but-may-increase-anxiety.html.

[124] Andersen T.O., Langstrup H., Lomborg S. (2020). Experiences with Wearable Activity Data during Self-Care by Chronic Heart Patients: Qualitative Study. J. Med. Internet Res..

[125] Zawn Villines L. (2020). Mood Tracker Apps_ Learn More About Some of the Best Options Here. Medical News Today. www.medicalnewstoday.com/articles/mood-tracker-app.

[126] Mendu S., Baee S. (2020). Redesigning the Quantified Self Ecosystem with Mental Health in Mind.

[127] Majumdar N. Quantified Self Detecting and Resolving Depression by Your Mobile Phone–Emberify Blog. https://emberify.com/blog/quantified-self-depression/.

[128] (2016). Projects Institute for Health Metrics and Evaluation. www.healthdata.org/projects.

[129] Cilliers L. (2019). Wearable devices in healthcare: Privacy and information security issues. Health Inf. Manag. J..

[130] Tawalbeh L., Muheidat F., Tawalbeh M., Quwaider M. (2020). IoT Privacy and Security: Challenges and Solutions. Appl. Sci..

[131] Kapoor V., Singh R., Reddy R., Churi P. Privacy Issues in Wearable Technology: An Intrinsic Review. Proceedings of the International Conference on Innovative Computing and Communication (ICICC-2020).

[132] Sankar R., Le X., Lee S., Wang D. (2013). Protection of data confidentiality and patient privacy in medical sensor networks. Implantable Sensor Systems for Medical Applications.

[133] Alrababah Z. Privacy and Security of Wearable Devices. December 2020. https://www.researchgate.net/publication/347558128_Privacy_and_Security_of_Wearable_Devices.

[134] Paul G., Irvine J. (2014). Privacy Implications of Wearable Health Devices. Proceedings of the 7th International Conference on Security of Information and Networks.

[135] Nguyen T., Gupta S., Venkatesh S., Phung D. (2016). Nonparametric discovery of movement patterns from accelerometer signals. Pattern Recognit. Lett..

[136] Ushmani A. (2019). Machine Learning Pattern Matching. J. Comput. Sci. Trends Technol..

[137] Pendlimarri D., Petlu P.B.B. (2010). Novel Pattern Matching Algorithm for Single Pattern Matching. Int. J. Comput. Sci. Eng..

[138] Sarkania V.K., Bhalla V.K. (2013). Android Internals. Int. J. Adv. Res. Comput. Sci. Softw. Eng..

[139] Mohammed M., Khan M.B., Bashie E.B.M. (2016). Machine Learning: Algorithms and Applications.

[140] Gmyzin D. (2017). A Comparison of Supervised Machine Learning Classification Techniques and Theory-Driven Approaches for the Prediction of Subjective Mental Workload Subjective Mental. Master’s Thesis.

[141] Osisanwo F.Y., Akinsola J.E.T., Awodele O., Hinmikaiye J.O., Olakanmi O., Akinjobi J. (2017). Supervised Machine Learning Algorithms: Classification and Comparison. Int. J. Comput. Trends Technol..

[142] Rajoub B. (2020). Supervised and unsupervised learning. Biomedical Signal Processing and Artificial Intelligence in Healthcare.

[143] Rani S., Babbar H., Coleman S., Singh A. (2021). An Efficient and Lightweight Deep Learning Model for Human Activity Recognition Using Smartphones. Sensors.

[144] Pietroni F., Casaccia S., Revel G.M., Scalise L. Methodologies for continuous activity classification of user through wearable devices: Feasibility and preliminary investigation. Proceedings of the 2016 IEEE Sensors Applications Symposium (SAS).

[145] Ben-gal I. (2014). Outlier detection Irad Ben-Gal Department of Industrial Engineering. Data Mining and Knowledge Discovery Handbook.

[146] Colpas P.A., Vicario E., De-La-Hoz-Franco E., Pineres-Melo M., Oviedo-Carrascal A., Patara F. (2020). Unsupervised Human Activity Recognition Using the Clustering Approach: A Review. Sensors.

[147] Hailat Z., Komarichev A., Chen X.-W. Deep Semi-Supervised Learning. Proceedings of the 2018 24th International Conference on Pattern Recognition (ICPR).

[148] Stikic M., Larlus D., Schiele B. Multi-graph Based Semi-supervised Learning for Activity Recognition. Proceedings of the 2009 International Symposium on Wearable Computers.

[149] Lee J., Bahri Y., Novak R., Schoenholz S.S., Pennington J., Sohl-Dickstein J. (2017). Deep neural networks as gaussian processes. arXiv.

[150] Xu H., Li L., Fang M., Zhang F. (2018). Movement Human Actions Recognition Based on Machine Learning. Int. J. Online Eng. (iJOE).

[151] Fu A., Yu Y. (2017). Real-Time Gesture Pattern Classification with IMU Data. http://stanford.edu/class/ee267/Spring2017/report_fu_yu.pdf.

[152] Bujari A., Licar B., Palazzi C.E. Movement pattern recognition through smartphone’s accelerometer. Proceedings of the 2012 IEEE Consumer Communications and Networking Conference (CCNC).

[153] Baca A. (2012). Methods for Recognition and Classification of Human Motion Patterns—A Prerequisite for Intelligent Devices Assisting in Sports Activities. IFAC Proc. Vol..

[154] Farhan H., Al-Muifraje M.H., Saeed T.R. (2020). A new model for pattern recognition. Comput. Electr. Eng..

[155] Harvey S., Harvey R. (2016). An introduction to artificial intelligence. Appita J..

[156] Neapolitan R.E., Jiang X. (2018). Neural Networks and Deep Learning.

[157] Maurer U., Smailagic A., Siewiorek D., Deisher M. Activity Recognition and Monitoring Using Multiple Sensors on Different Body Positions. Proceedings of the International Workshop on Wearable and Implantable Body Sensor Networks (BSN’06).

[158] Lara D., Labrador M.A. A mobile platform for real-time human activity recognition. Proceedings of the 2012 IEEE Consumer Communications and Networking Conference (CCNC).

[159] Tapia E.M., Intille S.S., Haskell W., Larson K., Wright J., King A., Friedman R. Real-time recognition of physical activities and theirintensities using wireless accelerometers and a heart monitor. Proceedings of the 2007 11th IEEE International Symposium on Wearable Computers.

[160] Tzu-Ping K., Che-Wei L., Jeen-Shing W. Development of a portable activity detector for daily activity recognition. Proceedings of the IEEE International Symposium on Industrial Electronics.

[161] Bhat G., Deb R., Ogras U.Y. (2019). OpenHealth: Open-Source Platform for Wearable Health Monitoring. IEEE Des. Test.

[162] Nakamura Y., Matsuda Y., Arakawa Y., Yasumoto K. (2019). WaistonBelt X:A Belt-Type Wearable Device with Sensing and Intervention Toward Health Behavior Change. Sensors.

[163] Munoz-Organero M. (2019). Outlier Detection in Wearable Sensor Data for Human Activity Recognition (HAR) Based on DRNNs. IEEE Access.

[164] Lara O.D., Labrador M.A. (2013). A Survey on Human Activity Recognition using Wearable Sensors. IEEE Commun. Surv. Tutor..

[165] Kaghyan S., Sarukhanyan H.G. (2012). Activity recognitionusing k-nearest neighbor algorithm on smartphone with triaxial accelerometer. Int. J. Inform. Models Anal..

[166] Arias P., Kelley C., Mason J., Bryant K., Roy K. Classification of User Movement Data. Proceedings of the 2nd International Conference on Digital Signal Processing.

[167] Seok W., Kim Y., Park C. (2018). Pattern Recognition of Human Arm Movement Using Deep Reinforcement Learning Intelligent Information System and Embedded Software Engineering.

[168] Gupta S.M., Mujawar A. Tracking and Prediciting Movement Patterns of a Moving Object in Wiresless Sensor Network. Proceedings of the 2018 2nd International Conference on Trends in Electronics and Informatics (ICOEI).

[169] Zhang Y., Zhang Z., Zhang Y., Bao J., Zhang Y., Deng H. (2019). Human Activity Recognition Based on Motion Sensor Using U-Net. IEEE Access.

[170] Xu C., Chai D., He J., Zhang X., Duan S. (2019). InnoHAR: A Deep Neural Network for Complex Human Activity Recognition. IEEE Access.

[171] Clouthier A.L., Ross G.B., Graham R.B. (2020). Sensor Data Required for Automatic Recognition of Athletic Tasks Using Deep Neural Networks. Front. Bioeng. Biotechnol..

[172] Hwang I., Cha G., Oh S. Multi-modal human action recognition using deep neural networks fusing image and inertial sensor data. Proceedings of the 2015 IEEE International Conference on Multisensor Fusion and Integration for Intelligent Systems (MFI).

[173] Gumaei A., Hassan M.M., Alelaiwi A., Alsalman H. (2019). A Hybrid Deep Learning Model for Human Activity Recognition Using Multimodal Body Sensing Data. IEEE Access.

[174] Karungaru S. Human action recognition using wearable sensors and neural networks. Proceedings of the 2015 10th Asian Control Conference (ASCC).

[175] Choi A., Jung H., Mun J.H. (2019). Single Inertial Sensor-Based Neural Networks to Estimate COM-COP Inclination Angle during Walking. Sensors.

[176] Xie B., Li B., Harland A. Movement and Gesture Recognition Using Deep Learning and Wearable-sensor Technology. Proceedings of the 2018 International Conference on Artificial Intelligence and Pattern Recognition.

[177] Nguyen T., Gupta S., Venkatesh S., Phung D. A Bayesian Nonparametric Framework for Activity Recognition Using Accelerometer Data. Proceedings of the 2014 22nd International Conference on Pattern Recognition.

[178] Cheng L., You C., Guan Y., Yu Y. Body activity recognition using wearable sensors. Proceedings of the 2017 Computing Conference.

[179] Chen Y., Guo M., Wang Z. An improved algorithm for human activity recognition using wearable sensors. Proceedings of the 2016 Eighth International Conference on Advanced Computational Intelligence (ICACI).

[180] Mekruksavanich S., Jitpattanakul A. Classification of Gait Pattern with Wearable Sensing Data. Proceedings of the 2019 Joint International Conference on Digital Arts, Media and Technology with ECTI Northern Section Conference on Electrical, Electronics, Computer and Telecommunications Engineering.

[181] Hachaj T., Piekarczyk M. (2019). Evaluation of Pattern Recognition Methods for Head Gesture-Based Interface of a Virtual Reality Helmet Equipped with a Single IMU Sensor. Sensors.

[182] Kim M., Cho J., Lee S., Jung Y. (2019). IMU Sensor-Based Hand Gesture Recognition for Human-Machine Interfaces. Sensors.

[183] DiLiberti N., Peng C., Kaufman C., Dong Y., Hansberger J.T. Real-Time Gesture Recognition Using 3D Sensory Data and a Light Convolutional Neural Network. Proceedings of the 27th ACM International Conference on Multimedia.

[184] Alavi S., Arsenault D., Whitehead A. (2016). Quaternion-Based Gesture Recognition Using Wireless Wearable Motion Capture Sensors. Sensors.

[185] Santhoshkumar R., Geetha M.K. (2019). Deep Learning Approach for Emotion Recognition from Human Body Movements with Feedforward Deep Convolution Neural Networks. Procedia Comput. Sci..

[186] Hu B., Dixon P.C., Jacobs J., Dennerlein J., Schiffman J. (2018). Machine learning algorithms based on signals from a single wearable inertial sensor can detect surface- and age-related differences in walking. J. Biomech..

[187] Lin W.-Y., Verma V.K., Lee M.-Y., Lai C.-S. (2018). Activity Monitoring with a Wrist-Worn, Accelerometer-Based Device. Micromachines.

[188] Estévez P.A., Held C.M., Holzmann C.A., Perez C.A., Pérez J.P., Heiss J., Garrido M., Peirano P. (2002). Polysomnographic pattern recognition for automated classification of sleep-waking states in infants. Med. Biol. Eng. Comput..

[189] Procházka A., Kuchyňka J., Vyšata O., Cejnar P., Vališ M., Mařík V. (2018). Multi-Class Sleep Stage Analysis and Adaptive Pattern Recognition. Appl. Sci..

[190] Gandhi R. (2018). Introduction to Machine Learning Algorithms: Linear Regression. Toward Data Science. https://towardsdatascience.com/introduction-to-machine-learning-algorithms-linear-regression-14c4e325882a.

[191] Duffy S.A. (2013). HHS Public Access Author manuscript. J. Community Health.

[192] Migueles J.H., Rowlands A.V., Huber F., Sabia S., Van Hees V.T. (2019). GGIR: A Research Community–Driven Open Source R Package for Generating Physical Activity and Sleep Outcomes from Multi-Day Raw Accelerometer Data. J. Meas. Phys. Behav..

[193] Kim Y., Hibbing P., Saint-Maurice P.F., Ellingson L.D., Hennessy E., Wolff-Hughes D.L., Perna F.M., Welk G.J. (2017). Surveillance of Youth Physical Activity and Sedentary Behavior with Wrist Accelerometry. Am. J. Prev. Med..

[194] Cole-kripke T., Daniel F., Sadeh T. (1992). ActiGraph White Paper Actigraphy Sleep Scoring Algorithms. https://actigraphcorp.com/.

[195] Quante M., Kaplan E.R., Cailler M., Rueschman M., Wang R., Weng J., Taveras E.M., Redline S. (2018). Actigraphy-based sleep estimation in adolescents and adults: A comparison with polysomnography using two scoring algorithms. Nat. Sci. Sleep.

[196] Haghayegh S., Khoshnevis S., Smolensky M.H., Diller K.R., Castriotta R.J. (2019). Performance comparison of different interpretative algorithms utilized to derive sleep parameters from wrist actigraphy data. Chrono. Int..

[197] Lee P.H., Suen L.K.P. (2016). The convergent validity of Actiwatch 2 and ActiGraph Link accelerometers in measuring total sleeping period, wake after sleep onset, and sleep efficiency in free-living condition. Sleep Breath.

